# Effects of Hydrostatic Pressure on Carcinogenic Properties of Epithelia

**DOI:** 10.1371/journal.pone.0145522

**Published:** 2015-12-30

**Authors:** Shinsaku Tokuda, Young Hak Kim, Hisako Matsumoto, Shigeo Muro, Toyohiro Hirai, Michiaki Mishima, Mikio Furuse

**Affiliations:** 1 Department of Respiratory Medicine, Graduate School of Medicine, Kyoto University, Kyoto, Japan; 2 Division of Cerebral Structure, National Institute for Physiological Sciences, Okazaki, Japan; University of Oslo, NORWAY

## Abstract

The relationship between chronic inflammation and cancer is well known. The inflammation increases the permeability of blood vessels and consequently elevates pressure in the interstitial tissues. However, there have been only a few reports on the effects of hydrostatic pressure on cultured cells, and the relationship between elevated hydrostatic pressure and cell properties related to malignant tumors is less well understood. Therefore, we investigated the effects of hydrostatic pressure on the cultured epithelial cells seeded on permeable filters. Surprisingly, hydrostatic pressure from basal to apical side induced epithelial stratification in Madin-Darby canine kidney (MDCK) I and Caco-2 cells, and cavities with microvilli and tight junctions around their surfaces were formed within the multi-layered epithelia. The hydrostatic pressure gradient also promoted cell proliferation, suppressed cell apoptosis, and increased transepithelial ion permeability. The inhibition of protein kinase A (PKA) promoted epithelial stratification by the hydrostatic pressure whereas the activation of PKA led to suppressed epithelial stratification. These results indicate the role of the hydrostatic pressure gradient in the regulation of various epithelial cell functions. The findings in this study may provide clues for the development of a novel strategy for the treatment of the carcinoma.

## Introduction

The relationship between chronic inflammation and cancer is well known [1]; epidemiological studies have shown that chronic inflammation predisposes individuals to various types of cancer, and inflammatory cells and inflammatory mediators, such as chemokines, cytokines, and transcription factors, have been reported to be involved in the pathways that link inflammation and cancer [2–4]. On the other hand, inflammation is also known to increase the permeability of blood vessels that subsequently elevates pressure in the interstitial tissues (Starling’s law of the capillaries), and elevated interstitial fluid pressure has been observed in most solid malignant tumors [5–8]. However, there have been only a few reports on the effects of hydrostatic pressure on cultured cells [6,8,9], and the relationship between elevated hydrostatic pressure and cell properties related to malignant tumors is less well understood [7].

In this study, we investigated the effects of hydrostatic pressure on cultured epithelial cells grown on permeable filters. Surprisingly, the hydrostatic pressure from basal to apical side induced epithelial stratification in Madin-Darby canine kidney (MDCK) I and Caco-2 cells. The effects of the hydrostatic pressure on various epithelial cell functions including cell polarity, cell proliferation and apoptosis, and transepithelial transport were further investigated in the study.

## Results and Discussion

### Hydrostatic pressure from basal to apical side triggers epithelial stratification in MDCK I cells

To investigate the effects of hydrostatic pressure on the epithelia, we seeded MDCK I cells (high-resistance strain of MDCK cells) at a density of 2 × 10^5^ cells/cm^2^ on Transwell permeable filters. Normally, the values of transepithelial electrical resistance (TER) in MDCK I cells at two days after seeding were higher than 1000 Ω·cm^2^, which indicated an establishment of confluent epithelial cell sheets. Then we varied the amounts of the culture medium in the apical and basal sides to apply hydrostatic pressure to MDCK I cell sheets at two days after seeding on filters (S1 Table). There was no air between epithelial cells and the culture medium. The culture medium was exchanged every two days, and the height of medium surfaces showed no apparent changes in the two days.

The intraluminal pressure in the urinary tract and gastrointestinal tract at resting phase is 5–15 cmH_2_O. In contrast, the interstitial fluid pressure in most normal tissues is tightly regulated and remains close to the atmospheric level (−4 to +4 cmH_2_O) [7]. Therefore, the pressure of several cmH_2_O from apical to basal side is thought to be applied to the epithelia under the normal condition. In contrast, the interstitial fluid pressure in a variety of human tumors is typically in the range of 14–54 cmH_2_O [7,10], thus the pressure of several cmH_2_O from basal to apical side is thought to be applied to the epithelia under these conditions. In this study, we examined the effects of the hydrostatic pressure of 0.6 cmH_2_O from basal to apical side (‘Basal’ condition) by comparing with that from apical to basal side (‘Apical’ condition) as a control experiment, although the in vivo situation of the hydrostatic pressure is more complex than the experimental conditions. The intensity of the hydrostatic pressure gradient (0.6 cmH_2_O) was determined considering the limitation in the height of the Transwell permeable filter.

**Fig 1:**
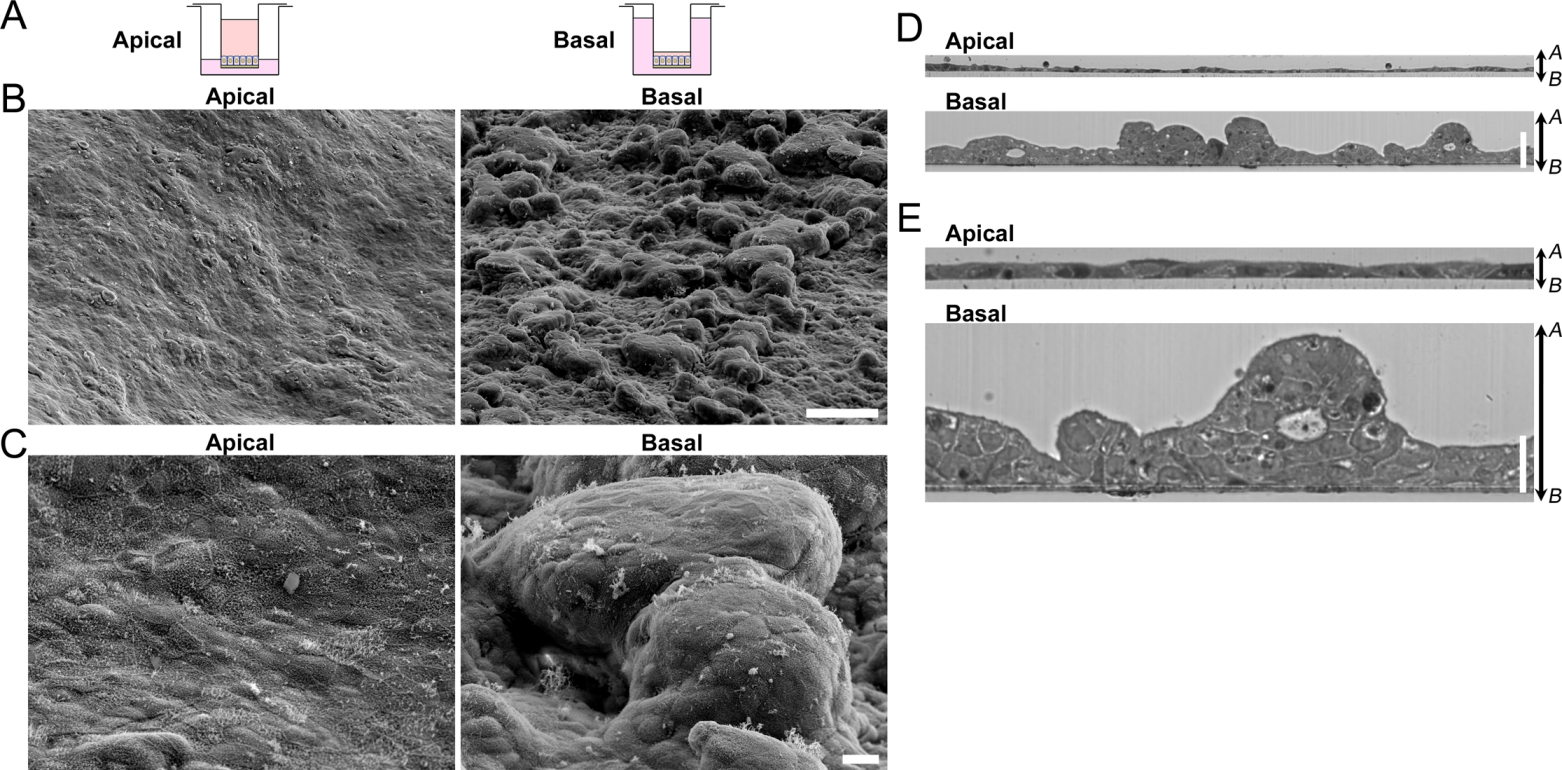
Scanning electron microscopy and light microscopic images in a vertical section of MDCK I cells under the ‘Apical’ and ‘Basal’ conditions. (A) Diagrams of culture conditions. MDCK I cells were seeded at a density of 2 × 10^5^ cells/cm^2^ on Transwell permeable filters, and the amounts of the culture medium in the apical and basal sides were varied at two days after seeding on filters. (B and C) Scanning electron micrographs of MDCK I cell sheets at low magnification (B) and high magnification (C) at four days after the culture under the ‘Apical’ and ‘Basal’ conditions. A rugged surface of the cell sheet was observed in the ‘Basal’ condition. (D and E) A vertical section of MDCK I cell sheets at low magnification (D) and high magnification (E). MDCK I cells were seeded on filters, and cultured under the ‘Apical’ and ‘Basal’ conditions for four days. A vertical section of cell sheets was observed by light microscopy staining with toluidine blue. A multi-layered cell sheet with a number of raised cell clumps was observed under the ‘Basal’ condition. Scale bars = 100 μm for (B), 10 μm for (C), 50 μm for (D) and 20 μm for (E). *A*, apical side; *B*, basal side.

First, we observed MDCK I cell sheets by scanning electron microscopy at four days after application of hydrostatic pressure (six days after seeding) (Fig 1). A flat surface of the cell sheet was observed under the ‘Apical’ condition in which the height of the medium surface in the apical side was approximately 6 mm higher than that of the basal side (S1 Table). In contrast, we found a remarkably rugged surface of the cell sheet under the ‘Basal’ condition in which the height of the medium surface in the basal side was approximately 6 mm higher than that of the apical side. Close observation at high magnification revealed that the rugged surface was composed of raised MDCK I cells (Fig 1C).

To investigate the internal structure of MDCK I cell sheets, we observed a vertical section of cell sheets under the ‘Apical’ and ‘Basal’ conditions by light microscopy staining with toluidine blue. Under the ‘Apical’ condition, a single layer of the cell sheet was observed (Fig 1D). In contrast, we found a multi-layered cell sheet with a number of raised cell clumps under the ‘Basal’ condition (Fig 1E). These results indicate that the ‘Basal’ condition triggers epithelial stratification in MDCK I cells.

**Fig 2:**
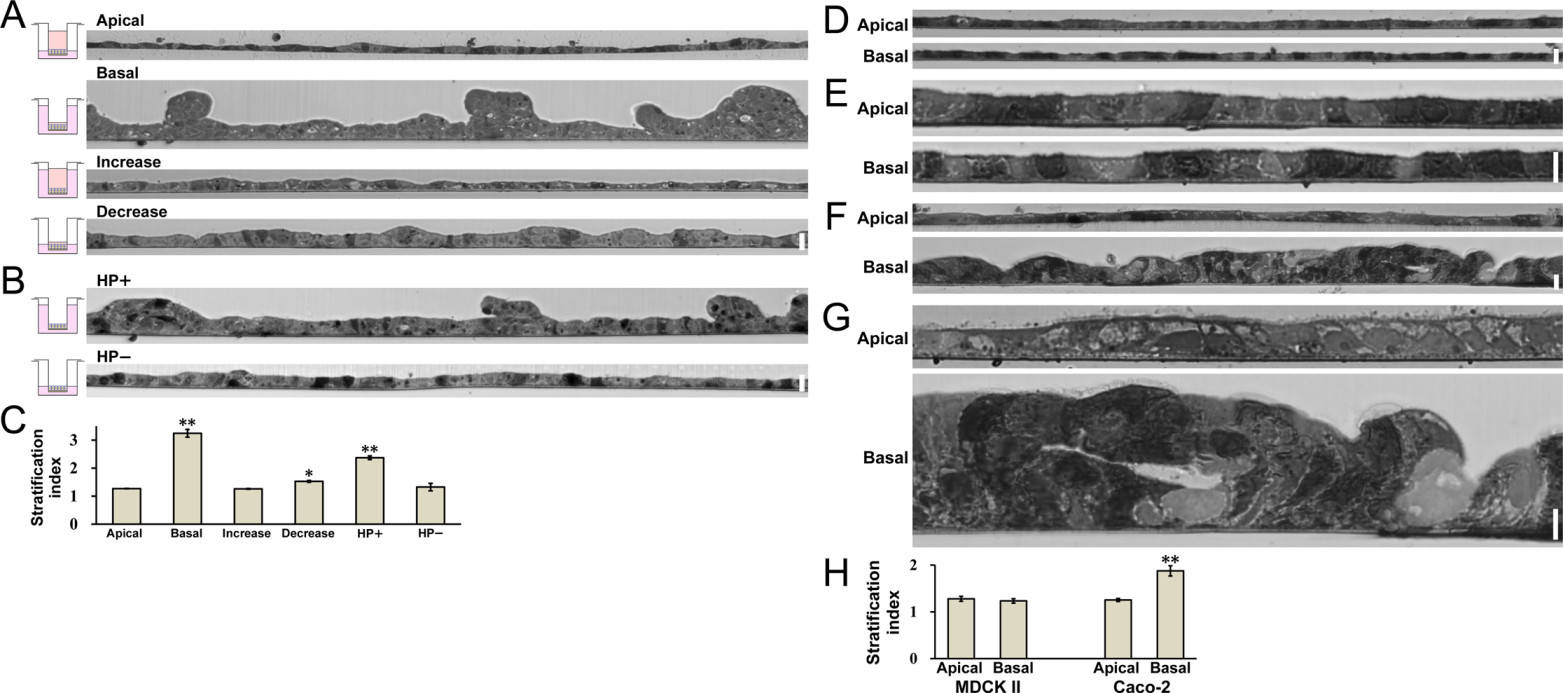
Light microscopic images in vertical sections of MDCK I cells, MDCK II cells and Caco-2 cells under the various hydrostatic pressure conditions. (A) A vertical section of MDCK I cell sheets. The culture medium in both the apical and basal sides was increased under the ‘Increase’ condition and decreased under the ‘Decrease’ condition. A slight degree of epithelial stratification was observed under the ‘Decrease’ condition. (B) A vertical section of MDCK I cell sheets. The culture medium in the apical side was almost eliminated and the hydrostatic pressure from basal to apical side was applied (‘HP+’ condition) or not applied (‘HP−’ condition) to the MDCK I cell sheets. Epithelial stratification was observed under the ‘HP+’ condition, whereas there was hardly any sign of epithelial stratification under the ‘HP−’ condition. (C) Stratification index under the conditions in (A) and (B). The degree of epithelial stratification (stratification index) was quantified as described in *Materials and Methods*. * *p* < 0.05, ** *p* < 0.01 compared with the ‘Apical’ condition. (D and E) A vertical section of MDCK II cell sheets at low magnification (D) and high magnification (E). MDCK II cells were seeded on filters, and cultured under the ‘Apical’ and ‘Basal’ conditions for four days. Epithelial stratification was not observed under the ‘Apical’ and ‘Basal’ conditions. (F and G) A vertical section of Caco-2 cell sheets at low magnification (F) and high magnification (G). Caco-2 cells were seeded on filters, and cultured under the ‘Apical’ and ‘Basal’ conditions for eight days. A multi-layered cell sheet was observed under the ‘Basal’ condition. (H) Stratification index in MDCK II and Caco-2 cells. ** *p* < 0.01 compared with the ‘Apical’ condition in corresponding cells. The upper side is apical side and the lower side is basal side. Scale bars = 20 μm for (A), (B), (D) and (F) and 10 μm for (E) and (G).

In the ‘Basal’ condition, the difference in the height of medium surfaces between the apical and basal sides is thought to act as hydrostatic pressure of 0.6 cmH_2_O from basal to apical side to the epithelial cell sheets, which is likely to cause epithelial stratification in MDCK I cells. However, the larger amount of the culture medium in the basal side (‘Basal’ condition, 2200μl vs ‘Apical’ condition, 1200μl) or the smaller amount of the culture medium in the apical side under the ‘Basal’ condition (‘Basal’ condition, 120μl vs ‘Apical’ condition, 960μl) also potentially contributes to the epithelial stratification in MDCK I cells. To examine the effects of the amount of culture medium on the epithelial stratification, MDCK I cells were cultured under the conditions in which the culture medium in both the apical and basal sides was either increased (‘Increase’ condition) or decreased (‘Decrease’ condition) (S1 Table). The vertical section of cell sheets was observed by light microscopy (Fig 2A and 2B). Under the ‘Increase’ condition, a single layer of the cell sheet was observed similar to that of the ‘Apical’ condition (Fig 2A), indicating that the increase in the culture medium in the basal side does not trigger epithelial stratification when the culture medium is not decreased in the apical side. In contrast, we found a slight degree of epithelial stratification under the ‘Decrease’ condition (Fig 2A). To quantitatively evaluate the degree of epithelial stratification, we counted the cell number on vertical lines drawn at 20 μm intervals on the vertical section of MDCK I cell sheets (stratification index; S1 Fig). The stratification index under the ‘Decrease’ condition was significantly higher than that under the ‘Apical’ condition (Fig 2C).

A small amount of culture medium in the apical side forms a concave meniscus because of the adhesion between the culture medium and the inner wall of the filter cup. The surface tension of the concave meniscus is thought to act as a physical force to pull up the culture medium, which may lead to epithelial stratification in MDCK I cells. To confirm the effects of the small amount of the culture medium in the apical side, we cultured MDCK I cells under the conditions in which the culture medium in the apical side was almost eliminated and the hydrostatic pressure from basal to apical side was applied (‘HP+’ condition) or not applied (‘HP−’ condition) to the MDCK I cell sheets (S1 Table). The vertical section of cell sheets was observed by light microscopy (Fig 2B). Under the ‘HP−’ condition, there was hardly any sign of epithelial stratification in the MDCK I cell sheet. In contrast, a multi-layered cell sheet was observed under the ‘HP+’ condition, although the stratification index was lower than that under the ‘Basal’ condition (Fig 2C). These results indicate that hydrostatic pressure from basal to apical side triggers epithelial stratification in MDCK I cells, and a small amount of culture medium in the apical side also causes a mild degree of epithelial stratification.

### Hydrostatic pressure from basal to apical side triggers epithelial stratification in Caco-2 cells but not in MDCK II cells

To investigate whether the epithelial stratification by the hydrostatic pressure is a phenomenon specific to MDCK I cells or not, we examined the effects of the hydrostatic pressure on epithelial stratification in MDCK II cells (low-resistance strain of MDCK cells) and Caco-2 cells (human colon carcinoma cells). Since the growth rate of Caco-2 cells was slower than that of MDCK cells, the hydrostatic pressure was applied for eight days in Caco-2 cells. In MDCK II cells, no apparent epithelial stratification was found under the ‘Apical’ and ‘Basal’ condition (Fig 2D, 2E and 2H). In contrast, a multi-layered cell sheet was observed in the case of Caco-2 cells under the ‘Basal’ condition (Fig 2F, 2G and 2H). These results indicate that the epithelial stratification induced by the hydrostatic pressure from basal to apical side is not a specific phenomenon to MDCK I cells and also occurs in Caco-2 cells, and responsiveness to the hydrostatic pressure varies depending on the cell types.

### Time course and reversibility of epithelial stratification by the hydrostatic pressure

**Fig 3:**
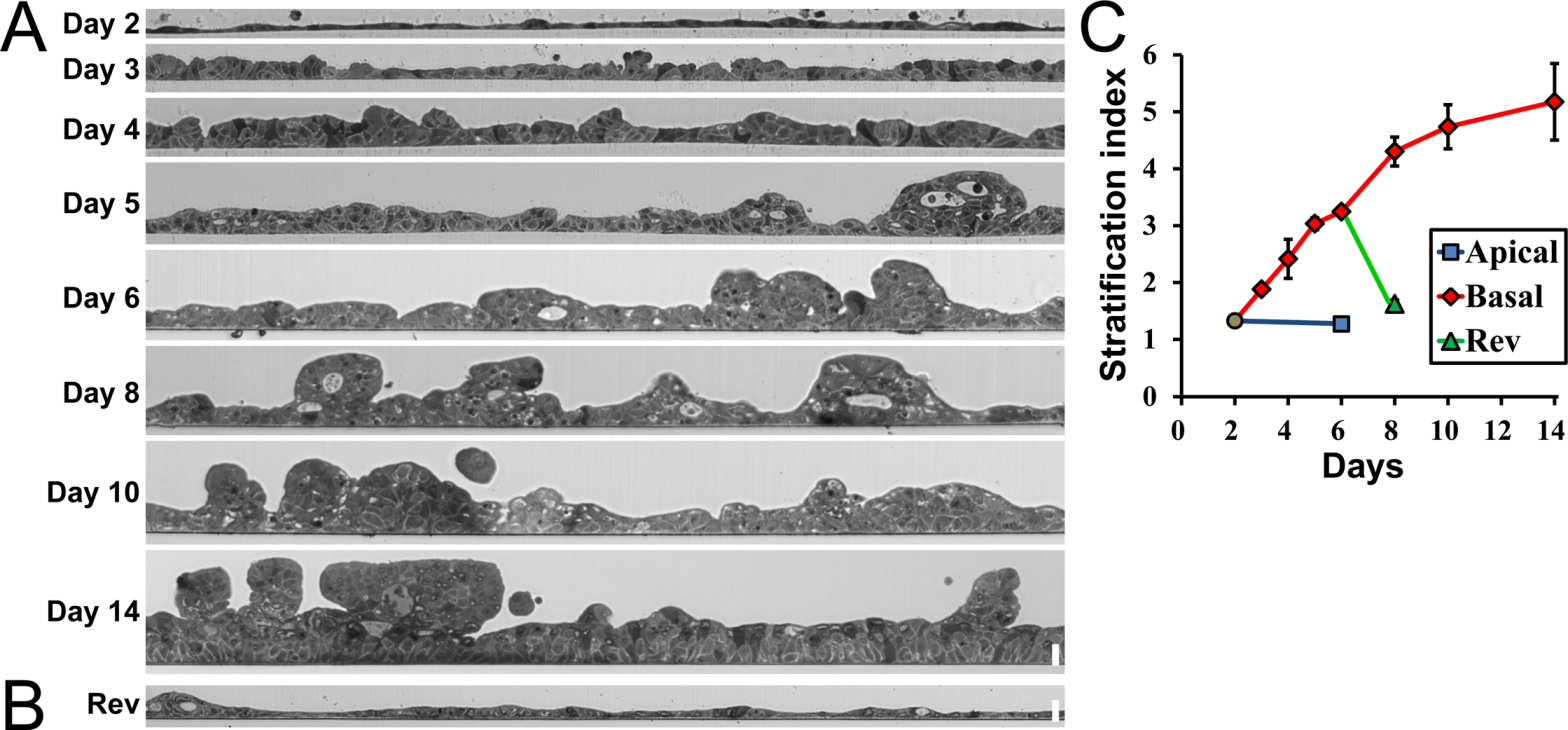
Time course and reversibility of epithelial stratification by the hydrostatic pressure. (A) A vertical section of MDCK I cell sheets. Hydrostatic pressure from basal to apical side was applied to the MDCK I cell sheets at two days after seeding on filters, and the vertical section of cell sheets was observed at Day 2 and 1–12 days after application of the hydrostatic pressure (Days 3–14). MDCK I cells showed gradual development of epithelial stratification with time. (B) The reversibility of epithelial stratification by the hydrostatic pressure. Hydrostatic pressure from basal to apical side was applied to the MDCK I cell sheets for four days, and the hydrostatic pressure gradient was then eliminated by the increase of the culture medium in the apical side. (C) Stratification index under the conditions in (A) and (B). The upper side is apical side and the lower side is basal side. Scale bars = 20 μm.

Next, we investigated the time course of epithelial stratification by the hydrostatic pressure in MDCK I cells. We applied hydrostatic pressure from basal to apical side to MDCK I cell sheets at two days after seeding on filters, and the vertical section of cell sheets was observed at Day 2 and 1–12 days after application of the hydrostatic pressure (Days 3–14) (Fig 3A). MDCK I cells showed gradual development of epithelial stratification with time, and the stratification index consistently increased for 12 days during the application of the hydrostatic pressure (Fig 3A and 3C).

We also examined the reversibility of epithelial stratification by the hydrostatic pressure. We applied hydrostatic pressure from basal to apical side to MDCK I cell sheets for four days, and the hydrostatic pressure gradient was then eliminated by the increase of the culture medium in the apical side. A single layer of the cell sheet with a few multi-layered regions was observed at two days after the elimination of the hydrostatic pressure gradient (Fig 3B and 3C), indicating the reversibility of the epithelial stratification by the hydrostatic pressure.

### Effects of the hydrostatic pressure on cell polarity

**Fig 4:**
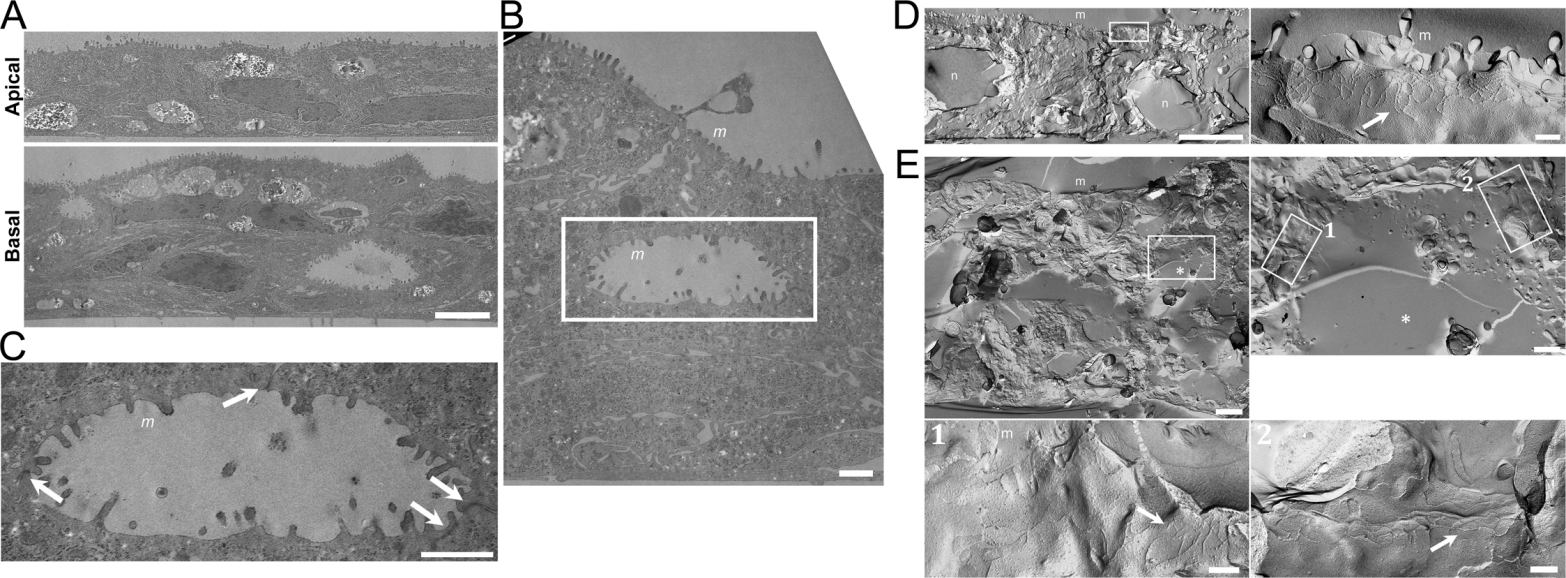
Transmission and freeze-fracture electron microscopy of MDCK I cells under the ‘Apical’ and ‘Basal’ conditions. (A) Transmission electron micrographs in a vertical section of MDCK I cell sheets at low magnification under the ‘Apical’ and ‘Basal’ conditions. MDCK I cells were observed at four days after the culture under the ‘Apical’ and ‘Basal’ conditions. Cavities were observed within the multi-layered MDCK I cells under the ‘Basal’ condition. Scale bar = 5 μm. (B) A transmission electron micrograph in a vertical section of MDCK I cell sheets at high magnification under the ‘Basal’ condition. The structure of microvilli was observed at the surfaces of the cavities and apical cell membranes of the outermost cell layer. Scale bar = 1 μm. (C) An enlarged image in the region enclosed by the white line in the transmission electron micrograph (B). Plasma membranes between the adjacent cells were fused immediately below the surface of the cavity (*arrows*). Scale bar = 1 μm. (D) Freeze-fracture electron micrographs in a lateral view of MDCK I cells under the ‘Apical’ condition at low magnification (left panel) and high magnification (right panel). TJ strands were observed immediately below the microvilli (*arrow*). Scale bars = 5 μm for the left panel and 200 nm for the right panel. (E) Freeze-fracture electron micrographs in a lateral view of MDCK I cells under the ‘Basal’ condition at low magnification (upper left panel), an enlarged image in the region enclosed by the white line in the upper left panel (upper right panel), and further enlarged images in the regions enclosed by the white lines in the upper right panel (lower panels). TJ strands were observed immediately below the surface of the cavity within the multi-layered MDCK I cells (*arrows*). Scale bars = 5 μm for the upper left panel, 1 μm for the upper right panel, and 200 nm for the lower panels. *m*, microvilli; *n*, nucleus; *, cavity.

For a detailed study on epithelial stratification by the hydrostatic pressure, we observed MDCK I cells by transmission electron microscopy at four days after the culture under the ‘Apical’ and ‘Basal’ conditions (Fig 4A–4C). Under the ‘Apical’ condition, we observed a single layer of MDCK I cells that had short and sparse microvilli at apical cell membranes, which was consistent with a previous study [11] (Fig 4A). In contrast, the epithelial stratification observed under the ‘Basal’ condition showed the presence of a number of cavities within the multi-layered cell sheets of MDCK I cells. Interestingly, we found the microvilli structures at the surfaces of the cavities as well as at apical cell membranes of the outermost cell layer. Close observation of the cavities at high magnification revealed that plasma membranes between the adjacent cells were fused immediately below the surface of the cavity (Fig 4B and 4C). Since the fusion of plasma membranes between the adjacent cells is the characteristic structure of tight junctions (TJs) in transmission electron micrographs [12], it was likely that TJs were formed between the adjacent cells surrounding the cavities within the multi-layered epithelia.

Epithelial cells adhere to each other through junctional complexes, and TJs are located in the most apical part of the complexes at the boundary between the apical and basolateral cell membranes [12,13]. In freeze-fracture electron micrographs, TJs appear as continuous anastomosing particle strands (TJ strands) [14]. To confirm whether the fusion of plasma membranes around the cavities observed in the transmission electron micrographs are TJs or not, we observed MDCK I cell sheets by freeze-fracture electron microscopy at four days after the culture under the ‘Apical’ and ‘Basal’ conditions (Fig 4D and 4E). Under the ‘Apical’ condition, TJ strands were observed immediately below the microvilli in the lateral view of MDCK I cells in freeze-fracture electron micrographs (Fig 4D). In contrast, we observed multi-layered MDCK I cells with a number of cavities under the ‘Basal’ condition, and TJ strands were occasionally found immediately below the surface of the cavity (Fig 4E). These results indicate that the cavities with microvilli and TJs around their surfaces are formed within the multi-layered epithelia induced by the hydrostatic pressure from basal to apical side in MDCK I cells. These results also suggest that the MDCK I cells surrounding the cavities have the property of apical-basal polarity with a surface of the cavity as an apical side.

### Localization of TJ proteins in the multi-layered epithelia induced by the hydrostatic pressure

**Fig 5:**
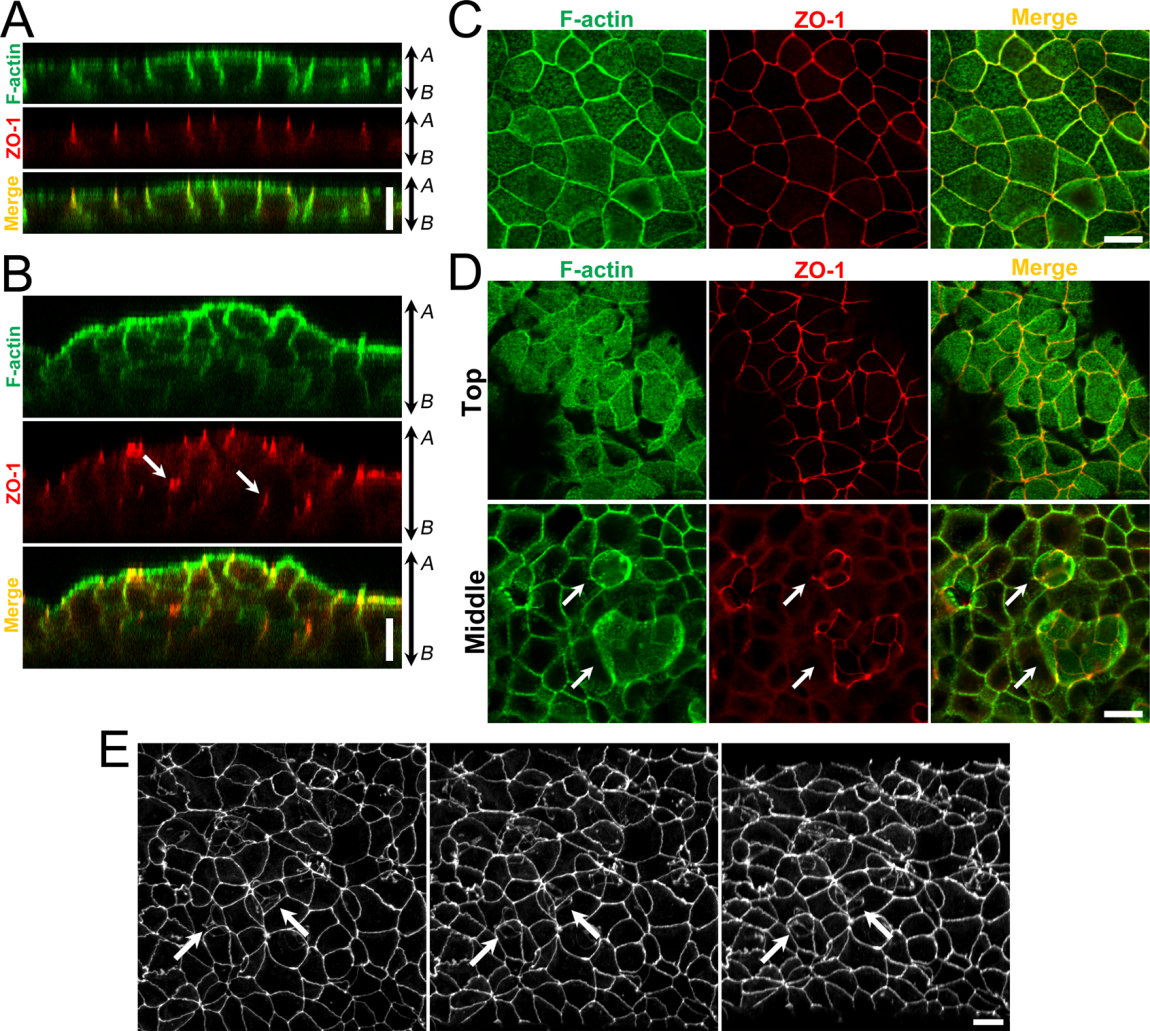
Localization of ZO-1 and F-actin under the ‘Apical’ and ‘Basal’ conditions in MDCK I cells. (A and B) Immunofluorescence microscopy for ZO-1 and F-actin in z-axis plane under the ‘Apical’ (A) and ‘Basal’ (B) conditions in MDCK I cells. The signals of ZO-1 were observed within the multi-layered MDCK I cells under the ‘Basal’ condition (*arrows*). *A*, apical side; *B*, basal side. (C and D) Immunofluorescence microscopy for ZO-1 and F-actin in xy plane under the ‘Apical’ (C) and ‘Basal’ (D) conditions in MDCK I cells. In the middle level of multi-layered MDCK I cells under the ‘Basal’ condition, the lines of ZO-1 signals were observed with spherical staining of F-actin (*arrows*). (E) Three-dimensional images of ZO-1 signals in the multi-layered MDCK I cells under the ‘Basal’ condition. ZO-1 signals under the ‘Basal’ condition in MDCK I cells were captured by confocal microscopy, and three-dimensional images were constructed from an integration of the confocal scanning images. Closed lines of ZO-1 signals were found within the multi-layered MDCK I cells (*arrows*). Scale bars = 10 μm.

**Fig 6:**
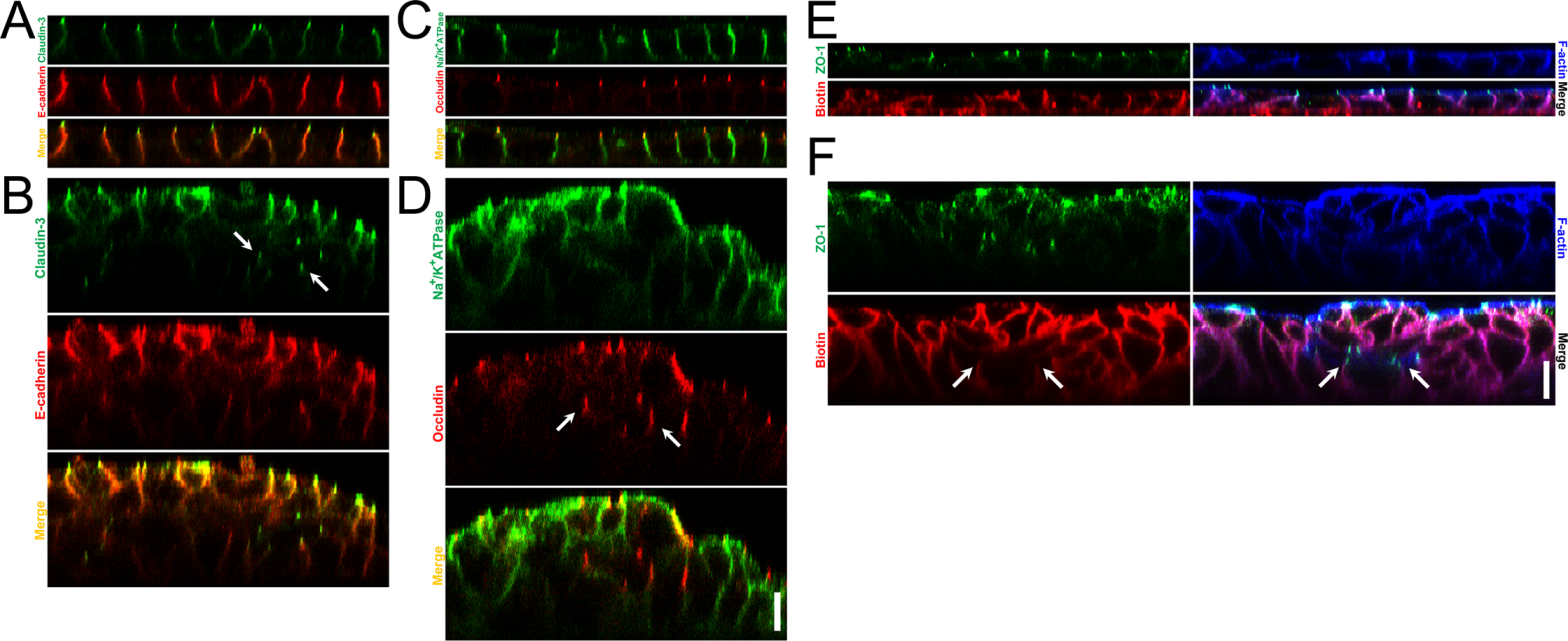
Localization of claudin-3, E-cadherin, occludin and Na^+^/K^+^ ATPase under the ‘Apical’ and ‘Basal’ conditions in MDCK I cells and barrier function of TJs around the cavities within the multi-layered epithelia. (A and B) Immunofluorescence microscopy for claudin-3 and E-cadherin in z-axis plane under the ‘Apical’ (A) and ‘Basal’ (B) conditions in MDCK I cells. Signals of claudin-3 were observed within the multi-layered MDCK I cells under the ‘Basal’ condition (*arrows*). (C and D) Immunofluorescence microscopy for occludin and Na^+^/K^+^ ATPase in z-axis plane under the ‘Apical’ (C) and ‘Basal’ (D) conditions in MDCK I cells. Signals of occludin were observed within the multi-layered MDCK I cells under the ‘Basal’ condition (*arrows*). (E and F) A tracer experiment using a primary amine-reactive biotinylation reagent was performed in MDCK I cells cultured under the ‘Apical’ (E) and ‘Basal’ (F) conditions. The biotinylation reagent was administered in the basal side for 10 min, and the bound biotin was detected by streptavidin. The biotinylation reagent appeared to stop at TJs around the cavities within the multi-layered epithelia under the ‘Basal’ condition (*arrows*). Scale bars = 10 μm.

Currently many proteins have been identified as TJ components such as a scaffold protein zonula occludens-1 (ZO-1) [15] and integral membrane proteins occludin [16] and claudins [17]. To confirm the localization of TJ proteins at cell-cell contacts around the cavities present within the multi-layered epithelia, we examined the localization of ZO-1 in MDCK I cells at four days after the culture under the ‘Apical’ and ‘Basal’ conditions by immunofluorescence microscopy (Fig 5). Under the ‘Apical’ condition, signals of ZO-1 were concentrated at the most apical regions of cell-cell contacts with the faint signals along the lateral membranes in MDCK I cells (Fig 5A), which was consistent with previous studies [18–20]. In contrast, signals of ZO-1 were found within the multi-layered epithelia as well as at cell-cell contacts of the outermost cell layer (Fig 5B). In three-dimensional images constructed from an integration of confocal scanning images, closed lines of ZO-1 signals were found within the multi-layered epithelia under the ‘Basal’ condition (Fig 5E and S1 Movie). Spherical staining of F-actin was also observed within the multi-layered epithelia under the ‘Basal’ condition (Fig 5D), which was thought to represent the signals of F-actin of microvilli observed in the electron microscopy. We also examined the localization of claudin-3 and occludin in MDCK I cells (Fig 6A–6D). Signals of claudin-3 and occludin were also observed within the multi-layered epithelia under the ‘Basal’ condition. These results indicate that TJ proteins including ZO-1, claudin(s) and occludin localize at the TJs in the MDCK I cells surrounding the cavities.

We also examined the localization of ZO-1 and F-actin in Caco-2 cells (S2 Fig). Signals of ZO-1 and spherical staining of F-actin were found within the multi-layered epithelia under the ‘Basal’ condition. These results suggest that the formation of the cavities with microvilli and TJs around their surfaces also occurs in Caco-2 cells within the multi-layered epithelia induced by the hydrostatic pressure from basal to apical side.

### Barrier function of the TJs around the cavities within the multi-layered epithelia

Epithelia act as a barrier to the external environment, and TJs are known to regulate the movements of substances through the paracellular pathway [21,22]. To investigate whether the TJs at cell-cell contacts around the cavities act as a barrier in the paracellular pathway, we performed a tracer experiment using a primary amine-reactive biotinylation reagent which labels proteins and primary amine-containing macromolecules on a cell surface [23]. We administered the biotinylation reagent into the basal solution for 10 min, and the bound biotin was detected by streptavidin. Under the ‘Apical’ condition, the biotinylation reagent appeared to diffuse along the intercellular space from the basal side but stop at the level of TJs represented by the concentrated ZO-1 signals (Fig 6E). In contrast, the biotinylation reagent not only stopped at TJs in the outermost cell layer but also stopped at the TJs around the cavities within the multi-layered epithelia under the ‘Basal’ condition (Fig 6F). These results indicate that the TJs at cell-cell contacts around the cavities within the multi-layered epithelia also show the function as the paracellular barrier.

### Effects of the hydrostatic pressure on cell proliferation

**Fig 7:**
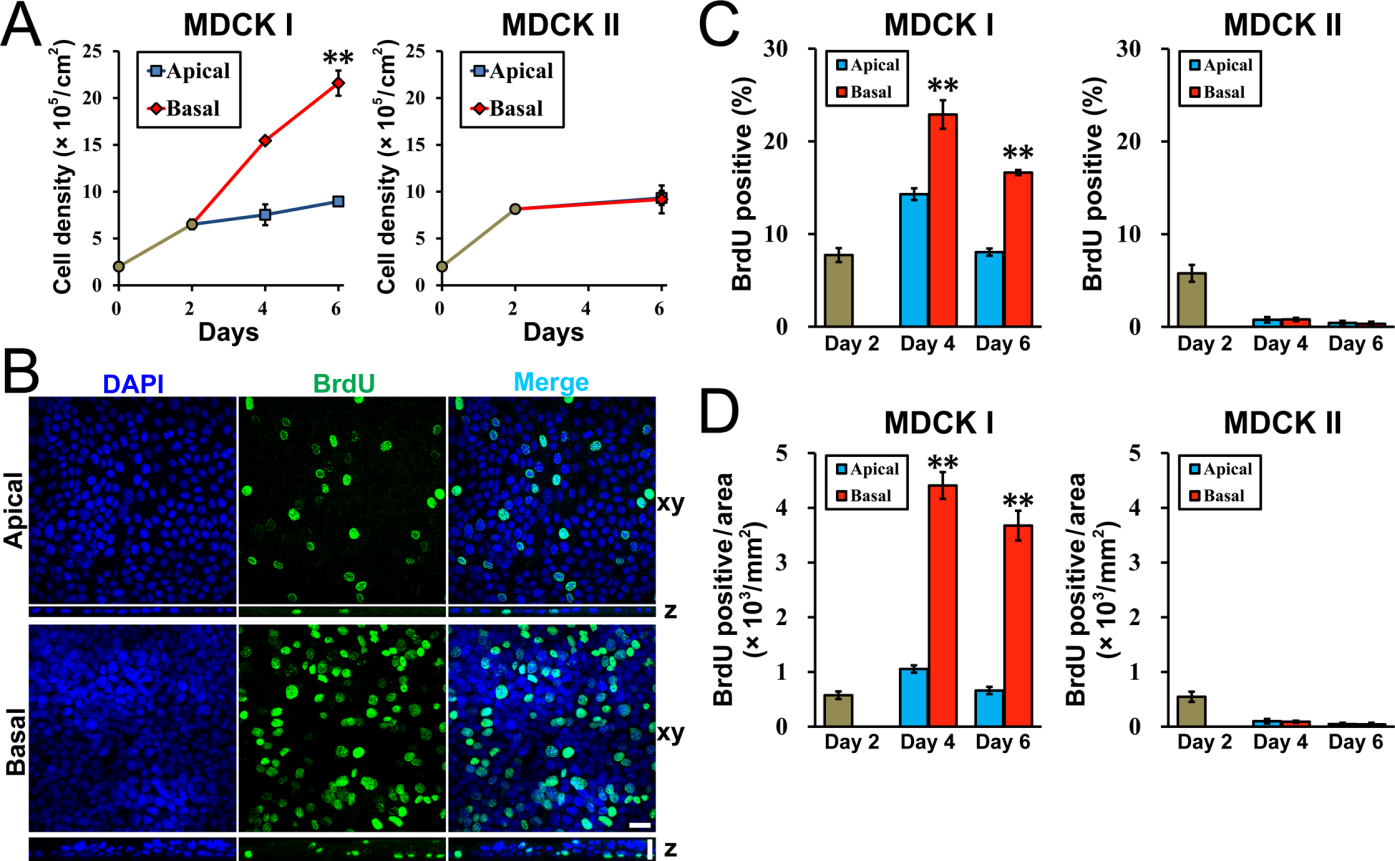
Effects of hydrostatic pressure on cell proliferation. (A) Effects of hydrostatic pressure on the cell number in MDCK I and MDCK II cells cultured on filters. MDCK I and MDCK II cells were seeded at a density of 2 × 10^5^ cells/cm^2^ on filters, and the cell number was counted with counting chamber after the trypsinization of the cells at each time point. The density of MDCK I cells under the ‘Basal’ condition was significantly higher than that under the ‘Apical’ condition. (B) 5-bromo-2’-deoxy-uridine (BrdU) assay under the ‘Apical’ and ‘Basal’ conditions in MDCK I cells. BrdU assay was performed as described in *Materials and Methods*. The upper side is apical side and the lower side is basal side in z axis plane. Scale bars = 20 μm. (C) The ratio of BrdU-positive cells in MDCK I and MDCK II cells cultured on filters. The ratio of the cell number of BrdU-positive cells to that of DAPI-positive cells was calculated. The ratio of BrdU-positive cells under the ‘Basal’ condition was significantly higher than that under the ‘Apical’ condition in MDCK I cells. (D) The density of BrdU-positive cells in MDCK I and MDCK II cells cultured on filters. The density of BrdU-positive cells under the ‘Basal’ condition was more than four-fold higher than that under the ‘Apical’ condition in MDCK I cells. ** *p* < 0.01 compared with the ‘Apical’ condition at corresponding days.

In the multi-layered epithelia, it is speculated that the total cell number in the filter cup is increased compared to that of the epithelia of single layer. To confirm this possibility, we trypsinized the cells on filters and counted the cell number with counting chamber under the ‘Apical’ and ‘Basal’ conditions in MDCK I and MDCK II cells. MDCK I cells seeded at a density of 2 × 10^5^ cells/cm^2^ on filters were increased to the density of 6.51 × 10^5^ cells/cm^2^ at two days after seeding on filters (Fig 7A). Under the ‘Apical’ condition, the density of MDCK I cells was additionally increased by 38% during the four days of the culture (8.95 × 10^5^ cells/cm^2^). In contrast, the density of MDCK I cells was additionally increased by 232% during the four days of the culture under the ‘Basal’ condition (21.60 × 10^5^ cells/cm^2^). The density of MDCK I cells at four days after the culture under the ‘Increase’ condition in which there was no difference of the height of the medium surface between the apical and basal sides was comparable to that under the ‘Apical’ condition (8.62 × 10^5^ cells/cm^2^) (S3 Fig). On the other hand, MDCK II cells seeded at a density of 2 × 10^5^ cells/cm^2^ on filters were increased to the density of 8.14 × 10^5^ cells/cm^2^ at two days after seeding, and the density was additionally increased by approximately 10% during the four days of the culture regardless of the ‘Apical’ or ‘Basal’ condition. These results indicate that the cell number show an increase by the hydrostatic pressure from basal to apical side in MDCK I cells but not in MDCK II cells, which is consistent with the hypothesis that the cell number is increased in the multi-layered epithelia.

Next, we performed 5-bromo-2’-deoxy-uridine (BrdU) assay to assess cell proliferation. We administered BrdU for 1 h, and the cells incorporating BrdU into their DNA in place of thymidine were detected by a monoclonal antibody against BrdU [24] (Fig 7B). Then we counted the total number of the cells stained with 4’, 6-diamidino-2-phenylindole (DAPI) and the number of BrdU-positive cells, and then calculated the ratio of the cell number of BrdU-positive cells to the total cell number. In MDCK I cells, the ratio of BrdU-positive cells was 7.73 ± 0.75% at two days after seeding, and the ratio was slightly increased at day 4 and recovered to the level of day 2 at day 6 (day 4 ‘Apical’, 14.30 ± 0.64%; day 6 ‘Apical’, 8.05 ± 0.39%) (Fig 7C). In contrast, the ratio of BrdU-positive cells under the ‘Basal’ condition was significantly higher than that of the ‘Apical’ condition (day 4 ‘Basal’, 22.90 ± 1.53%; day 6 ‘Basal’, 16.63 ± 0.27%). These results indicate that hydrostatic pressure from basal to apical side promotes cell proliferation in MDCK I cells. We also calculated the number of BrdU-positive cells per unit area since the density of MDCK I cells under the ‘Basal’ condition was thought to be higher than that of the ‘Apical’ condition. The density of BrdU-positive cells under the ‘Basal’ condition was more than four-fold higher than that under the ‘Apical’ condition at days 4 and 6 in the case of MDCK I cells (Fig 7D), suggesting that the increase of proliferating cells under the ‘Basal’ condition is likely to contribute to the increase of the cell number in MDCK I cells.

On the other hand, the ratio of BrdU-positive cells was 5.78 ± 0.91% at two days after seeding in MDCK II cells, and the ratio fell to less than 1% on days 4 and 6 under the ‘Apical’ and ‘Basal conditions (Fig 7C) (day 4 ‘Apical’, 0.77 ± 0.30%; day 4 ‘Basal’, 0.80 ± 0.18%; day 6 ‘Apical’, 0.43 ± 0.22%; day 6 ‘Basal’, 0.33 ± 0.22%). The tendency of culture cells to stop or slow their proliferation as they grow to a confluence is known as contact inhibition [25], and MDCK II cells have been reported to exhibit the ability of contact inhibition [26]. The decrease in the ratio of BrdU-positive cells after day 4 is thought to reflect the contact inhibition in MDCK II cell, and the hydrostatic pressure from basal to apical side is likely to have no significant effect on cell proliferation in MDCK II cells.

### Effects of the hydrostatic pressure on cell apoptosis

**Fig 8:**
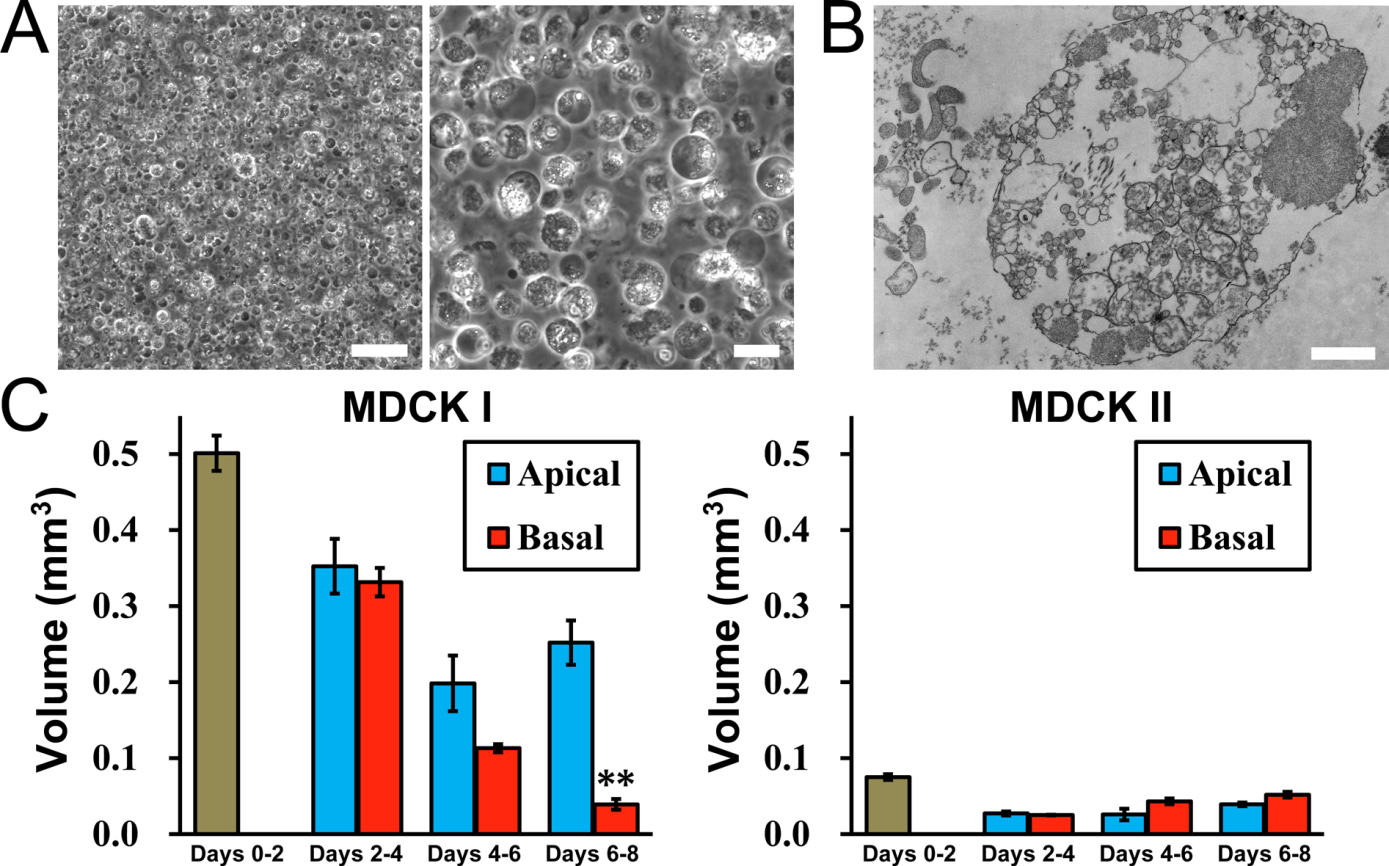
Effects of hydrostatic pressure on cell death. (A) Phase-contrast microscopic images of the culture supernatant of MDCK I cells cultured on filters for four days. Many spherical floating objects were observed. Scale bars = 100 μm for left panel and 20 μm for right panel. (B) A transmission electron micrograph of the culture supernatant of MDCK I cells. Scale bar = 1 μm. (C) Volume of the debris in the culture supernatant. The volume of the debris was measured as described in *Materials and Methods*. The volume of debris in days 6–8 under the ‘Basal’ condition was significantly smaller than that under the ‘Apical’ condition in MDCK I cells. ** *p* < 0.01 compared with the ‘Apical’ condition at corresponding day.

We also investigated the effects of the hydrostatic pressure on cell death. To confirm the occurrence of cell death in MDCK I cells, we first observed the culture supernatant of MDCK I cells cultured on filters for four days by phase-contrast microscopy. Many spherical floating objects were observed in the culture supernatant of MDCK I cells (Fig 8A). In transmission electron microscopy, a lot of small vesicles and fragmented nuclei were observed in the culture supernatant of MDCK I cells (Fig 8B). Then we evaluated the volume of the debris in the culture supernatant. We collected the culture supernatant at every two days at the exchange of the culture medium, and the supernatant was put into the microcapillary tubes with 0.29 mm inner diameter and centrifuged to pack the debris (S4 Fig). The packed debris was observed by light microscopy, and we confirmed that the packed debris was composed of dead cells (S4 Fig). Then the height of the packed debris in the microcapillary tubes was measured, and the volume of the dead cells was calculated as an area of the tube × a height of the debris. In MDCK I cells, the volume of the dead cells in the culture supernatant collected at day 2 was 0.501 ± 0.023 mm^3^. The volume was slightly decreased during days 2–4, days 4–6 and days 6–8, but it was approximately half of the volume in days 0–2 under the ‘Apical’ condition (days 2–4 ‘Apical’, 0.352 ± 0.036 mm^3^; days 4–6 ‘Apical’, 0.198 ± 0.038 mm^3^; days 6–8 ‘Apical’, 0.252 ± 0.029 mm^3^). In contrast, the volume of the dead cells was gradually decreased under the ‘Basal’ condition. The volume in days 6–8 under the ‘Basal’ condition was significantly smaller than that under the ‘Apical’ condition (days 2–4 ‘Basal’, 0.331 ± 0.019 mm^3^; days 4–6 ‘Basal’, 0.113 ± 0.005 mm^3^; days 6–8 ‘Basal’, 0.039 ± 0.007 mm^3^). These results suggest that hydrostatic pressure from basal to apical side suppresses the occurrence of cell death in MDCK I cells. On the other hand, the volume of the dead cells in the culture supernatant collected at day 2 in MDCK II cells was approximately 15% of that in MDCK I cells (0.075 ± 0.004 mm^3^). The volume was further decreased after day 2 regardless of the ‘Apical’ or ‘Basal’ condition, indicating a low frequency of the occurrence of cell death in MDCK II cells cultured on filters (Fig 8C).

**Fig 9:**
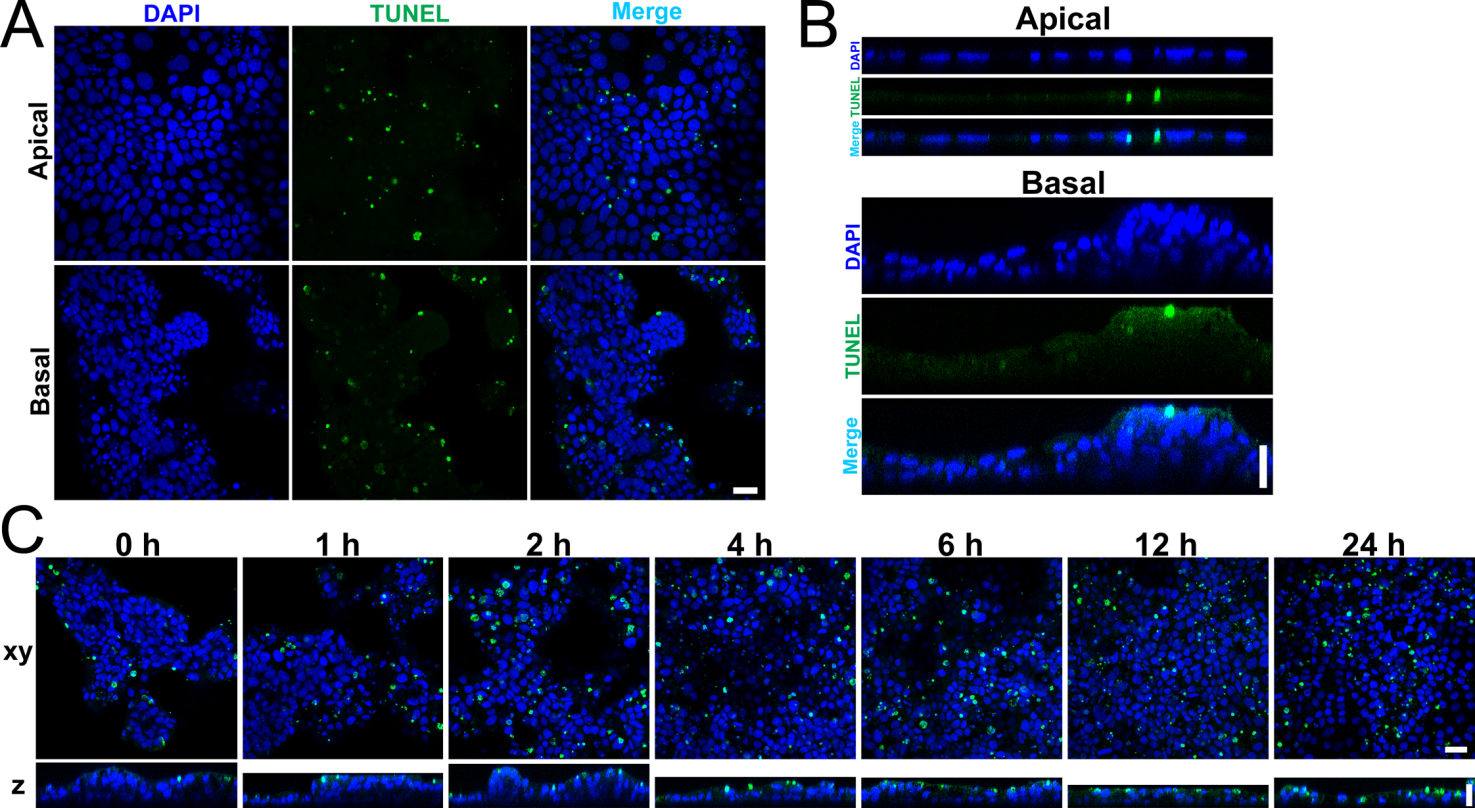
Terminal deoxynucleotidyl transferase-dUTP nick end labeling (TUNEL) assay. (A and B) TUNEL assay at four days after the culture under the ‘Apical’ and ‘Basal’ conditions in MDCK I cells in xy plane (A) and z-axis plane (B). (C) TUNEL assay after the elimination of the hydrostatic pressure gradient in MDCK I cells. MDCK I cells were cultured in the ‘Basal’ condition for four days, and the hydrostatic pressure gradient was eliminated by the increase of the culture medium in the apical side. TUNEL assay was performed at 0–24 h after the elimination of the hydrostatic pressure gradient. The fluorescent signals of fragmented DNA were obviously increased from 6 h after the elimination of the hydrostatic pressure gradient. The upper side is apical side and the lower side is basal side in z axis plane. Scale bars = 20 μm.

The fragmented nuclei observed in the transmission electron micrograph of the culture supernatant of MDCK I cells are one of the characteristic findings in apoptotic cells [27], although the packed debris may also include the dead cells due to causes other than apoptosis. To confirm whether the cell apoptosis is suppressed by the hydrostatic pressure from basal to apical side in MDCK I cells, we conducted terminal deoxynucleotidyl transferase-dUTP nick end labeling (TUNEL) assay that detect fragmented DNA [28]. We performed TUNEL assay at four days after the culture under the ‘Apical’ and ‘Basal’ conditions in MDCK I cells. The density of fluorescent signals of DNA fragmentation under the ‘Basal’ condition was slightly lower than that under the ‘Apical’ condition in MDCK I cells (Fig 9A and 9B and S4 Fig). Since each apoptotic cell generates a number of fluorescent signals of fragmented DNA, it was thought to be difficult to accurately quantify the occurrence of cell apoptosis by TUNEL assay and assess the difference between the ‘Apical’ and ‘Basal’ conditions. Therefore, we performed TUNEL assay after the elimination of the hydrostatic pressure from basal to apical side in MDCK I cells, and examined the occurrence of cell apoptosis during the decrease of epithelial stratification. We cultured MDCK I cells under the ‘Basal’ condition for four days, and investigated the time course of the occurrence of cell apoptosis after the elimination of the hydrostatic pressure gradient (Fig 9C). The fluorescent signals of fragmented DNA were slightly increased from 2 h after the elimination of the hydrostatic pressure gradient, and the signals were obviously increased from 6 h after the elimination of the hydrostatic pressure gradient accompanied by the decrease in the epithelial stratification (Fig 9C and S4 Fig). These results suggest that hydrostatic pressure from basal to apical side suppresses cell apoptosis in MDCK I cells.

### Effects of the hydrostatic pressure on transepithelial transport

**Fig 10:**
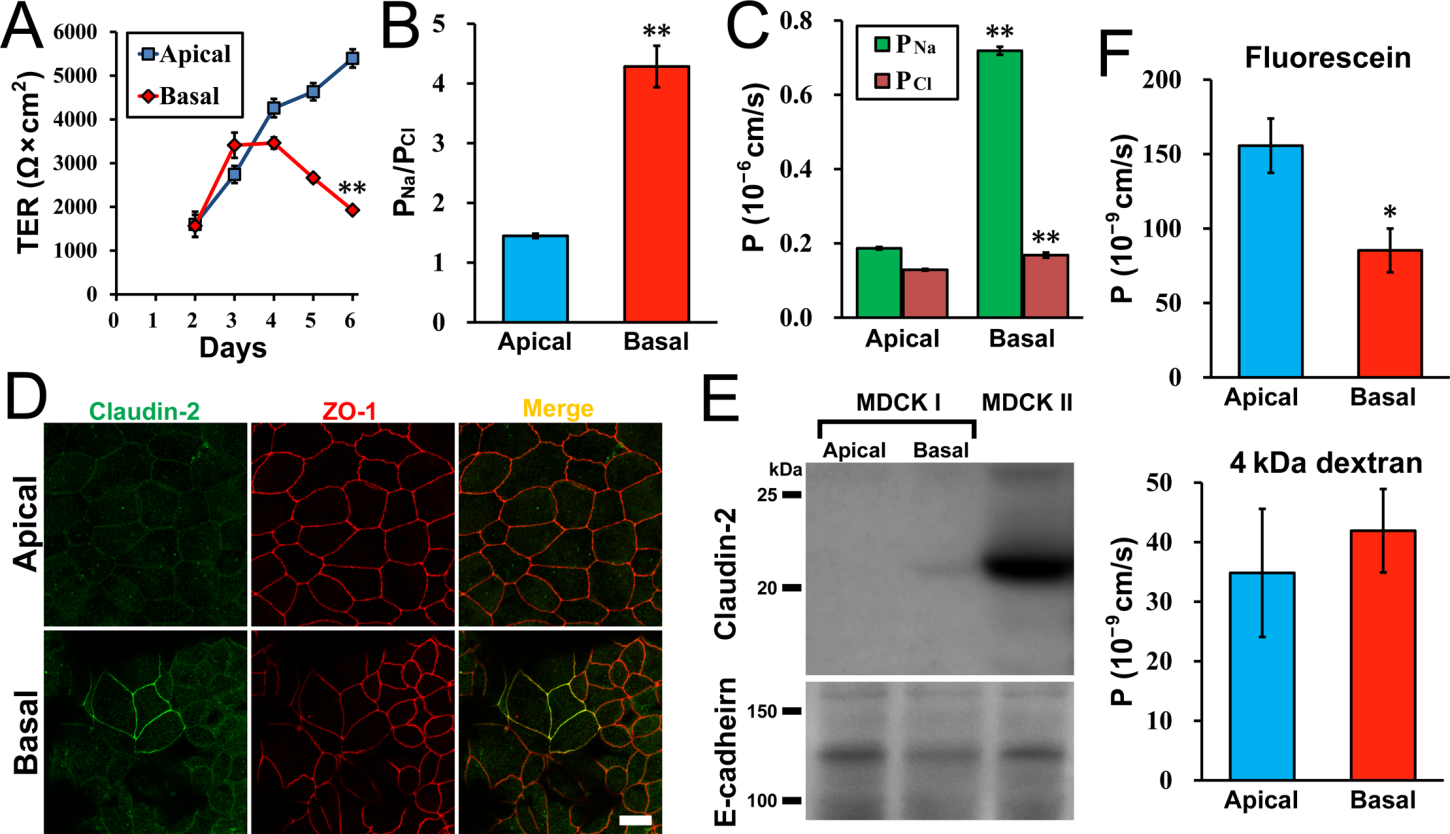
Effects of hydrostatic pressure on transepithelial transport. (A) Effects of hydrostatic pressure on transepithelial electrical resistance (TER) in MDCK I cells. The TER was changed to decrease at two days after application of the hydrostatic pressure under the ‘Basal’ condition. (B) The ratio of *P*_Na_ to *P*_Cl_ (*P*_Na_/*P*_Cl_) under the ‘Apical’ and ‘Basal’ conditions in MDCK I cells. The *P*_Na_/*P*_Cl_ was measured at four days after the culture under the ‘Apical’ and ‘Basal’ conditions. The value of *P*_Na_/*P*_Cl_ under the ‘Basal’ condition was significantly higher than that under the ‘Apical’ condition. (C) *P*_Na_ and *P*_Cl_ under the ‘Apical’ and ‘Basal’ conditions in MDCK I cells. The value of *P*_Na_ under the ‘Basal’ condition was approximately four-fold higher than that under the ‘Apical’ condition. (D) Immunofluorescence microscopy for claudin-2 and ZO-1 in MDCK I cells at four days after the culture under the ‘Apical’ and ‘Basal’ conditions. Claudin-2 staining was clearly detected at cell-cell contacts in a limited region under the ‘Basal’ condition. Scale bar = 10 μm. (E) Immunoblots for claudin-2 and E-cadherin in MDCK I cells at four days after the culture under the ‘Apical’ and ‘Basal’ conditions. MDCK II cells cultured on filters for six days were used as a positive control. A faint band of claudin-2 was detected under the ‘Basal’ condition in MDCK I cells. (F) Flux of fluorescein and 4 kDa FITC-dextran under the ‘Apical’ and ‘Basal’ conditions in MDCK I cells. The flux was measured at four days after the culture under the ‘Apical’ and ‘Basal’ conditions. The flux of fluorescein under the ‘Basal’ condition was significantly lower than that under the ‘Apical’ condition. * *p* < 0.05, ** *p* < 0.01 compared with the ‘Apical’ condition.

Transepithelial transport is one of the most important functions in the epithelia. To investigate the effects of the hydrostatic pressure on transepithelial transport, we measured TER, a reciprocal of the ion conductance across the epithelia under the ‘Apical’ and ‘Basal’ conditions in MDCK I cells (Fig 10A). Under the ‘Apical’ condition, the TER was gradually increased with time after seeding on filters, which was consistent with previous studies [11,19]. In contrast, the TER was similarly increased but later decreased at two days after the culture under the ‘Basal’ condition. The TER value at day 6 (four days after the culture under the ‘Basal’ condition) was approximately one third of that under the ‘Apical’ condition (‘Apical’, 5395 ± 210 Ω·cm^2^ vs ‘Basal’, 1924 ± 54 Ω·cm^2^). The TER under the ‘Increase’ condition in which there was no difference of the height of the medium surface between the apical and basal sides was comparable to that under the ‘Apical’ condition (S5 Fig).

Then, we measured the dilution potentials to examine Na^+^ and Cl^-^ permeability across the epithelia (*P*_Na_ and *P*_Cl_) at four days after the culture under the ‘Apical’ and ‘Basal’ conditions. The ratio of *P*_Na_ to *P*_Cl_ (*P*_Na_/*P*_Cl_) was 1.45 ± 0.04 under the ‘Apical’ condition in MDCK I cells, showing a slightly cation selective property of MDCK I cells consistent with a previous study [29] (Fig 10B). In contrast, MDCK I cells under the ‘Basal’ condition showed higher cation selectivity (*P*_Na_/*P*_Cl_, 4.28 ± 0.35). The value of *P*_Na_ under the ‘Basal’ condition was approximately four-fold higher than that under the ‘Apical’ condition (‘Apical’, 0.187 ± 0.004 × 10^−6^ cm/s vs ‘Basal’, 0.719 ± 0.011 × 10^−6^ cm/s) whereas the value of *P*_Cl_ under the ‘Basal’ condition was only slightly higher than that under the ‘Apical’ condition (‘Apical’, 0.129 ± 0.002 × 10^−6^ cm/s vs ‘Basal’, 0.168 ± 0.007 × 10^−6^ cm/s). These results suggest that the hydrostatic pressure from basal to apical side increases transepithelial ion permeability with a selective increase of *P*_Na_ than *P*_Cl_.

Transepithelial ion movements occur via two routes: transcellular and paracellular pathways. An active ion transport in the transcellular pathway generates transepithelial electrical potentials (Vt). In the so-called ‘tight’ epithelia which have high TER values such as MDCK I cells, the Vt generated by the active transport has significant effects on the dilution potentials that determine *P*_Na_/*P*_Cl_ [30,31]. Therefore, we measured Vt across MDCK I cell sheets in the culture medium under the ‘Apical’ and ‘Basal’ conditions (S5 Fig). The Vt showed small values regardless of the ‘Apical’ or ‘Basal’ condition in MDCK I cells (‘Apical’, 0.23 ± 0.09 mV; ‘Basal’, 0.00 ± 0.10 mV), suggesting that the effects of the active transport on dilution potentials were negligible in MDCK I cells. For further examination of the involvement of the transcellular pathway in the ion transport in MDCK I cells, we measured *P*_Na_ and *P*_Cl_ before and 30 min after the application of benzamil (epithelial sodium channel inhibitor), bumetanide (Na^+^-K^+^-Cl^-^ cotransporter inhibitor) and ouabain (Na^+^/K^+^-ATPase inhibitor) under the ‘Apical’ and ‘Basal’ conditions. The values of *P*_Na_ and *P*_Cl_ showed small changes by the application of the inhibitors regardless of the ‘Apical’ or ‘Basal’ condition (S5 Fig). These results suggest that the transcellular pathway is less involved in the increase of *P*_Na_ by the hydrostatic pressure from basal to apical side in MDCK I cells.

TJs regulate the movement of substances through the paracellular pathway [21,22], and the major determinants of TJ permeability are claudins, a large family of integral membrane proteins [17,32,33]. Since claudin-2 is known to form high conductive pores with cation selectivity in TJs [29,34,35], we evaluated the expression of claudin-2 by immunofluorescence microscopy. Under the ‘Apical’ condition, we did not find claudin-2 staining at cell-cell contacts, which was consistent with previous studies [19,29]. In contrast, claudin-2 staining was clearly detected at cell-cell contacts in some regions under the ‘Basal’ condition (Fig 10D and S6 Fig). Then immunoblot analysis was conducted for claudin-2. MDCK II cells were used as a positive control since they inherently express claudin-2 [19,34]. In MDCK I cells, the band of claudin-2 was not detected under the ‘Apical’ condition, which was consistent with previous studies [19,29], whereas a faint band of claudin-2 was detected under the ‘Basal’ condition (Fig 10E). Since a small amount of claudin-2 has been reported to have significant effects on *P*_Na_ [29,36], a trace amount of claudin-2 under the ‘Basal’ condition potentially contributes to the increase of *P*_Na_ by the hydrostatic pressure in MDCK I cells. We also investigated the effects of the hydrostatic pressure on transepithelial transport in MDCK II cells. The TER showed slightly lower values under the ‘Basal’ condition compared with ‘Apical’ condition, but the values of *P*_Na_/*P*_Cl_, *P*_Na_ and *P*_Cl_ under the ‘Basal’ condition were comparable to those under the ‘Apical’ condition (S7 Fig).

In the paracellular pathway, the permeability of larger molecules (> 4 Å) is separately regulated from the permeability of small molecules such as Na^+^ and Cl^-^ [31,37]. To examine the effects of the hydrostatic pressure on the permeability of larger molecules, we measured the flux of fluorescein (332 Da), a divalent midsized anion, and 4 kDa FITC-dextran in MDCK I cells at four days after the culture under the ‘Apical’ and ‘Basal’ conditions. The flux of fluorescein under the ‘Basal’ condition was lower than that under the ‘Apical’ condition, whereas the flux of 4 kDa FITC-dextran was not affected by the hydrostatic pressure (Fig 10F). These results suggest that the hydrostatic pressure from basal to apical side may decrease the permeability of larger molecules in the paracellular pathway. These results are also consistent with the previous study in which the overexpression of claudin-2 increased cation permeability without affecting the permeability of large molecules (> 4 Å) in TJs [37], although different mechanisms may be involved in the transepithelial transport in the multi-layered epithelia such as the transport via discontinuous cell-cell contacts and transcytosis observed in the endothelia [38]. Since the expression of claudins has been reported to be altered in numerous carcinomas, the expression of claudin-2 in MDCK I cells under the ‘Basal’ condition may be related to the alteration of claudin expression in carcinomas [39].

### Involvement of cell signaling in the epithelial stratification by the hydrostatic pressure

Finally, we investigated the involvement of cell signaling in the epithelial stratification by the hydrostatic pressure; we screened a role of common signal transduction mediators including protein kinase A (PKA), protein kinase C (PKC), and protein tyrosine kinase (PTK) using competitive inhibitors targeting the ATP binding sites of PKA (H89), PKC (Go6983), and PTK (genistein) in the epithelial stratification. Since the involvement of PKA in the epithelial stratification was suggested in the following experimental results, we also investigated the effects of the decrease in the intracellular levels of cyclic adenosine monophosphate (cAMP), a second messenger involved in the activation of PKA, by specific adenylyl cyclase inhibitor SQ22536, and the increase in the intracellular levels of cAMP by adenylyl cyclase activator forskolin and phosphodiesterase inhibitor 3-Isobutyl-1-methylxanthine (IBMX) on the epithelial stratification.

**Fig 11:**
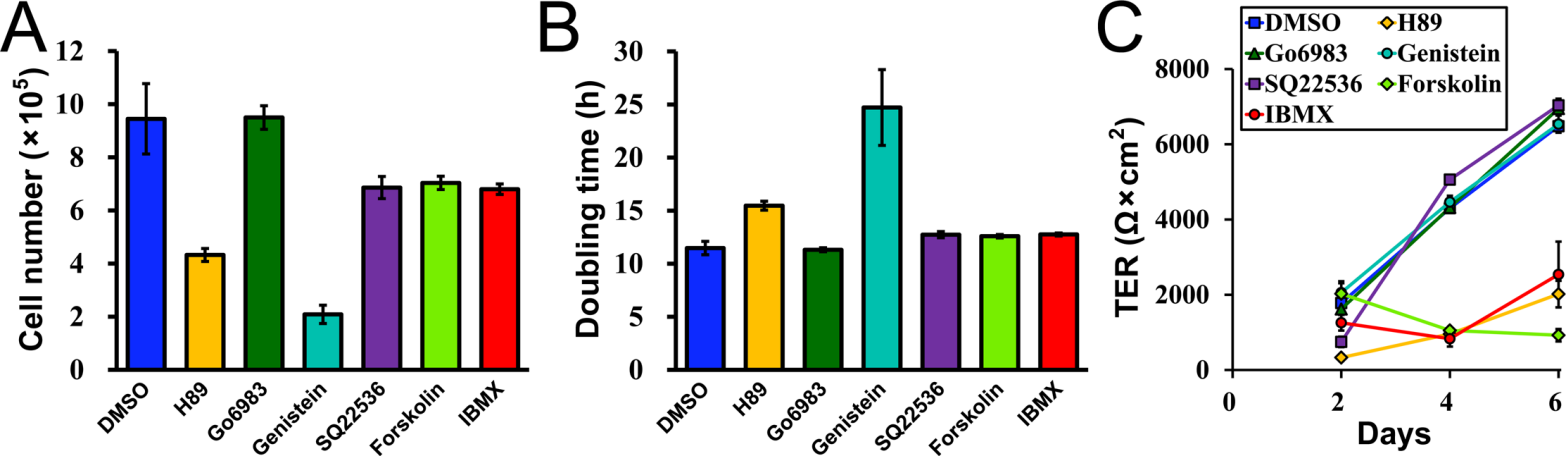
Effects of signal inhibitors and activators on cell proliferation and TER. (A) Effects of signal inhibitors and activators on the cell number in MDCK I cells. MDCK I cells were seeded at a density of 5.0 × 10^4^ cells/well in a 12-well plate, cultured for 48 h in the presence of the inhibitor or activator, and the cell number was counted with counting chamber after the trypsinization of the cells. (B) Effects of signal inhibitors and activators on the doubling time of MDCK I cells. The doubling time was calculated from the results in (A) on the assumption that the rate of cell proliferation was constant during the 48 h of the culture. (C) Effects of signal inhibitors and activators on TER. MDCK I cells were cultured on filters in the presence of the signal inhibitor or activator, and the TER was measured at each time point.

We first examined the effects of signal inhibitors and activators used in this study on cell proliferation. We seeded MDCK I cells at a density of 5.0 × 10^4^ cells/well in a 12-well plate and cultured for 48 h in the presence of the inhibitor or activator. Then the cell number was counted with the counting chamber (Fig 11A), and the doubling time was calculated on the assumption that the rate of cell proliferation was constant during the 48 h of culture (Fig 11B). The doubling time of MDCK I cells calculated under the control experiment (DMSO) was 11.5 h, and the administration of genistein extended the doubling time by 13.2 h, H89 extended by 4 h, and SQ22536, forskolin, and IBMX extended by approximately 1 h. Since both the inhibition of PKA (H89 and SQ22536) and the activation of PKA (forskolin and IBMX) extended the doubling time of MDCK I cells, the right level of PKA activity may contribute to the optimal cell proliferation in MDCK I cells. We also measured TER in the presence of the inhibitor or activator. The TER showed lower values in the presence of H89, forskolin, and IBMX (Fig 11C), but the values recorded at day 2 was still more than 300 Ω·cm^2^. Furthermore, the height of the medium surfaces showed no apparent changes in the presence of the signal inhibitor or activator under the ‘Apical’ and ‘Basal’ conditions in the following experiments.

**Fig 12:**
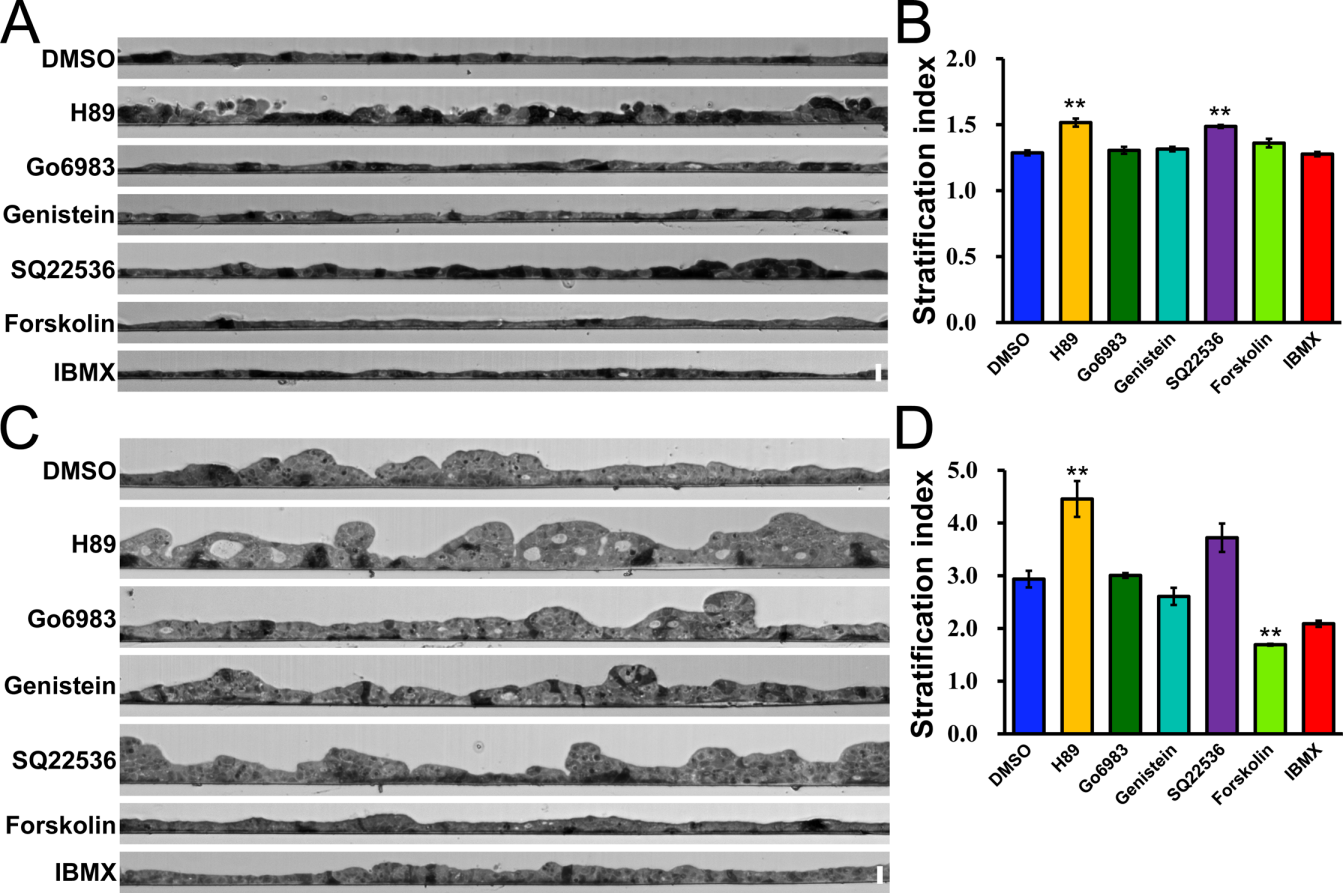
Effects of signal inhibitors and activators on the epithelial stratification by the hydrostatic pressure. (A) Light microscopic images in a vertical section under the ‘Apical’ condition in the presence of the signal inhibitor or activator in MDCK I cells. The upper side is apical side and the lower side is basal side. (B) Stratification index of MDCK I cells under the ‘Apical’ condition in the presence of the signal inhibitor or activator. The stratification index in the presence of H89 and SQ22536 was significantly higher than that in the control experiment (DMSO). (C) Light microscopic images in a vertical section under the ‘Basal’ condition in the presence of the signal inhibitor or activator in MDCK I cells. The upper side is apical side and the lower side is basal side. (D) Stratification index of MDCK I cells under the ‘Basal’ condition in the presence of the signal inhibitor or activator. The administration of H89 significantly increased the degree of epithelial stratification by the hydrostatic pressure, and the administration of forskolin significantly decreased the degree of the epithelial stratification. ** *p* < 0.01 compared with control (DMSO).

Then we investigated the effects of the inhibitors and activators on the epithelial stratification by the hydrostatic pressure. We seeded MDCK I cells on filters, and two days after seeding, we applied hydrostatic pressure in the presence of the inhibitor or activator. Surprisingly, the administration of H89, a PKA inhibitor, induced a slight degree of epithelial stratification even under the ‘Apical’ condition (Fig 12A and 12B). The administration of SQ22536, which decreases the intracellular levels of cAMP and results in the inhibition of PKA, also induced a slight degree of epithelial stratification under the ‘Apical’ condition. In contrast, a single layer of MDCK I cell sheet was observed in the presence of Go6983, genistein, forskolin and IBMX similar to the control experiment (DMSO) under the ‘Apical’ condition (Fig 12A and 12B).

Under the ‘Basal’ condition, the administration of H89 further increased the degree of epithelial stratification by the hydrostatic pressure (Fig 12C and 12D). The administration of SQ22536 also showed a tendency to increase the degree of epithelial stratification, although the difference in the stratification index did not reach a level of statistical significance compared with the control (DMSO). Since the activity of PKA is directly inhibited by H89 whereas the inhibition of PKA by SQ22536 is mediated by the decreases in the intracellular levels of cAMP, the difference between the stratification index in the presence of H89 and that of SQ22536 is likely to be due to the difference in the level of PKA inhibition by H89 and SQ22536. However, since the administration of H89 is also known to inhibit kinases other than PKA at high concentration, the non-specific effects of H89 also potentially caused the difference between the stratification index in the presence of H89 and SQ22536. Further analysis is required to understand the effects of PKA inhibition on epithelial stratification by the hydrostatic pressure. In contrast, the administration of forskolin, which increases the intracellular levels of cAMP and results in the activation of PKA, significantly decreased the degree of epithelial stratification under the ‘Basal’ condition. The administration of IBMX, which also increases the intracellular levels of cAMP in a different manner from forskolin, also showed a tendency to decrease the degree of epithelial stratification under the ‘Basal’ condition. These results suggest that the inhibition of PKA promotes epithelial stratification by the hydrostatic pressure whereas the activation of PKA suppresses the epithelial stratification in MDCK I cells.

In summary, this study shows that hydrostatic pressure gradient plays a role in the regulation of various epithelial cell functions: hydrostatic pressure from basal side to apical side induces epithelial stratification accompanied by the aberrant cell polarity, promotes cell proliferation, suppresses cell apoptosis, and increases transepithelial ion permeability. However, many questions are still unanswered in relation to this phenomenon. How do epithelia sense the hydrostatic pressure gradient? How does the process of epithelial stratification develop? What mechanisms are involved in the regulation of various cell functions by the hydrostatic pressure? Why does the responsiveness to the hydrostatic pressure gradient differ among different cell types? The involvement of the PKA signaling pathway in the epithelial stratification by the hydrostatic pressure observed in this study may offer a clue for the elucidation of the mechanisms of this phenomenon. Since the changes induced by the hydrostatic pressure from basal to apical side in the epithelial cells are likely to be related to the feature in the carcinoma [40], understanding of this phenomenon may contribute to the development of a novel strategy for the treatment of the carcinoma in future studies.

## Materials and Methods

### Cell culture

MDCK I cells provided by the late Dr. Shoichiro Tsukita (Kyoto University) [19], MDCK II cells provided by Dr. Masayuki Murata [19], and Caco-2 cells obtained from American Tissue Culture Collection have been maintained in our laboratory. MDCK I and II cells were grown in DMEM (high glucose) supplemented with 5% fetal bovine serum. For the culture of Caco-2 cells, 100 mm plastic dishes and Transwell permeable filters were coated with collagen (Cellmatrix Type I-C; Nitta Gelatin) following the manufacturer’s protocol. Caco-2 cells were grown in DMEM (high glucose) supplemented with 10% fetal bovine serum. In order to apply the hydrostatic pressure to the cell sheet cultured on the filters, the culture medium in the apical side was removed by the aspiration, and accurate amounts of the culture medium shown in S1 Table was added to the apical and basal sides by a pipette. The culture medium was exchanged every two days, and the height of the medium surfaces showed no apparent changes within the two days in all experiments in this study.

### Antibodies and reagents

Mouse anti-ZO-1 monoclonal antibody (mAb) (T8/754), rat anti-occludin mAb (MOC37), and rabbit anti-claudin-2 polyclonal antibody (pAb) were characterized as described previously [41–43]. Mouse anti-claudin-2 mAb (32–5600), rabbit anti-claudin-3 pAb (34–1700), alexa fluor 488 phalloidin (A12379) and alexa fluor 647 phalloidin (A22287) were purchased from Life Technologies. Mouse anti-E-cadherin mAb (ECCD-2; M108) was purchased from Clontech. Mouse anti-Na^+^/K^+^ ATPase α1 mAb (NB300-146) was purchased from Novus Biologicals. Fluorescein isothiocyanate-dextran (FITC-dextran), benzamil (B2417), bumetanide (B3023), ouabain (O3125), H89 (B1427), genistein (G6649), Go6983 (G1918) and 3-isobutyl-1-methylxanthine (IBMX, I5879) were purchased from Sigma Aldrich. Fluorescein (16106–82) was purchased from Nacalai tesque. SQ22536 was purchased from Santa Cruz. Forskolin was purchased from Wako.

### Transmission electron microscopy

Epithelial cells were plated at a density of 2 × 10^5^ cells/cm^2^ and grown on 12-mm diameter Transwell polycarbonate filters with a 0.4-µm pore size (Corning). After the culture under the ‘Apical’ and ‘Basal’ conditions for four days, the epithelia were fixed with 2% paraformaldehyde, 1% glutaraldehyde and 2% tannic acid/0.1 M HEPES buffer for 2 h at room temperature. Then the epithelia were rinsed thrice with HEPES buffer. The epithelia were post-fixed with 1% osmium tetroxide/0.1 M HEPES buffer for 60 min at 4°C. After washing with deionized water, the epithelia were dehydrated using increasing concentrations of ethanol (65%, 75%, 85%, 95%, 99%, and 100% for 10–15 min each). The ethanol was replaced with propylene oxide followed by the embedment of the epithelia in Poly/Bed 812 epoxy resin (Polyscience). The epithelia were sectioned at 70 nm using a diamond knife, and ultrathin sections were collected on 200-mesh copper grids followed by their staining with 1% hafnium chloride in methanol for 1 min, and subsequently by Sato’s lead citrate for 1 min. The samples were observed by a JEM-1011 transmission electron microscope (JEOL).

### Scanning electron microscopy

Epithelia grown on filters under the ‘Apical’ and ‘Basal’ conditions were fixed with 10% paraformaldehyde and 1% glutaraldehyde/0.1 M HEPES buffer for 2 h, post-fixed with 1% osmium tetroxide/0.1 M HEPES buffer for 60 min, and dehydrated with ethanol in a similar manner as described in *Transmission electron microscopy*. The ethanol was then replaced with t-butanol, and the epithelia were frozen at −20°C followed by the sublimation of t-butanol. The filters were mounted on aluminum planchets with carbon adhesive and coated with platinum using an ion coater, and the samples were observed by a JSM-6510LVS scanning electron microscope (JEOL).

### Light microscopy and stratification index

Epithelia grown on filters were fixed with 2% paraformaldehyde, 1% glutaraldehyde and 5% tannic acid/0.1 M HEPES buffer for 48–72 h. They were post-fixed with 1.8% osmium tetroxide/0.1 M HEPES buffer for 60 min, dehydrated with ethanol followed by the replacement with propylene oxide and embedded in epoxy resin in a similar manner as described in *Transmission electron microscopy*. The epithelia were sectioned at 3000 nm, and the sections were collected on a glass slide followed by staining with toluidine blue. The samples were observed by a light microscope (IX71; Olympus) and digital images were acquired using a cooled charge-coupled device camera (ORCAER; Hamamatsu Photonics K.K.). To quantitatively evaluate the degree of epithelial stratification, we counted the cell number on vertical lines drawn at 20 μm intervals on the vertical section of the epithelial cell sheets (S1 Fig). More than 140 lines were analyzed per sample, and the mean value of the cell number on the lines was defined as stratification index. N = 3–4 for each experiment.

### Freeze-fracture electron microscopy

Freeze-fracture electron microscopy was performed as described previously [19]. In brief, epithelia grown on 23-mm diameter Nunc polycarbonate filters with a 0.4-μm pore size (Life Technologies) were fixed with 2% paraformaldehyde and 2.5% glutaraldehyde/0.1 M phosphate buffer (pH 7.3) overnight. Then the epithelia were frozen in liquid nitrogen, and the frozen samples were fractured at −110°C and platinum-shadowed in Balzers Freeze Etching system (BAF060, Bal-Tec). Samples were observed by a JEM-1011 transmission electron microscope.

### Immunocytochemistry

Immunocytochemistry was performed as described previously [24]. In brief, epithelia grown on 12-mm filters were fixed with 10% paraformaldehyde for 7 min at room temperature or in 100% methanol for 10 min at −20°C. The filters were then permeabilized, in a solution of 0.2% (w/v) Triton X-100 (EMD Biosciences), blocked with 2% bovine serum albumin, and incubated with a primary Ab followed by a secondary Ab. The samples were imaged on a Zeiss LSM700 confocal microscope.

### BrdU assay

BrdU assay was performed using BrdU Labeling and Detection Kit I following the manufacturer’s protocol (Roche, 1296736). In brief, 10 μM BrdU was administered in a culture medium for 60 min, and the epithelia were fixed with 92% ethanol containing 50mM glycine (pH 2.0) for 20 min at −20°C. Then the filters were incubated with anti-BrdU mAb containing nucleases and were treated with fluorescein-labeled secondary Ab and DAPI. The samples were imaged on a Zeiss LSM700 confocal microscope, and nine randomly selected areas of 7730μm^2^ were analyzed per sample. The cell number of DAPI-positive cells and BrdU-positive cells were counted for each area, and the ratio of the cell number of BrdU-positive to DAPI-positive cells was calculated. N = 3–4 for each experiment.

### TUNEL assay

TUNEL assay was performed using Click-iT TUNEL Alexa Fluor 488 Imaging Assay following the manufacturer’s protocol (Life Technologies, C10245). In Brief, epithelia grown on 12-mm filters were fixed with 10% paraformaldehyde for 7 min, and permeabilized with Triton X-100. The modified dUTPs were incorporated by terminal deoxynucleotidyl transferase at the 3’-OH ends of fragmented DNA, a hallmark of apoptosis [28]. The modified dUTPs were detected by copper (I) catalyzed reaction between an alkyne in the modified dUTP and alexa fluor 488 azide. The density of TUNEL signals was quantified in a similar manner as described in *BrdU assay*.

### Quantification of the debris in the culture supernatant

The culture supernatant was collected at the exchange of the culture medium into the microtubes. After the centrifugation, pellets of the debris were resuspended in a small amount of PBS, which were put into the microcapillary tubes with 0.29 mm inner diameter (BRAND GMBH + CO KG, 708707). One end of the microcapillary tubes were clogged by putty, and the tubes were centrifuged to pack the debris (S4 Fig). The packed debris in the microcapillary tubes were captured by a stereomicroscope MZ10F (Leica) with an object micrometer, and the digital images were acquired using a cooled charge-coupled device camera (Rolera EM-C EMCCD camera; QImaging). The height of packed debris in the microcapillary tubes was measured using ImageJ 1.43u (available at http://rsb.info.nih.gov/ij; developed by Wayne Rasband, National Institutes of Health, Bethesda, MD), and the volume of the debris was calculated as: Area of the tube × Height of the debris. N = 4–6 for each experiment.

### Immunoblotting

Epithelial cells cultured on filters were scraped into Laemmli SDS sample buffer and boiled for 5 min. The proteins were separated by one-dimensional SDS-PAGE and electrotransferred from the gels to PVDF membranes followed by the incubation with primary Abs. The bound Abs were detected using HRP-linked secondary Abs and visualized by enhanced chemiluminescence (ECL Prime Kit; GE Healthcare).

### Electrophysiological measurements

Electrophysiological studies were performed as described previously [36,44]. In brief, electrical resistance and potentials across the epithelia were measured using Millicell-ERS epithelial volt-ohm meter (Millipore), and transepithelial electrical resistance (TER) and potentials (Vt) were determined by the subtraction of the resistance and potential of a blank filter. To determine the ion permeability of Na^+^ (*P*_Na_) and Cl^−^ (*P*_Cl_) across the epithelia, dilution potentials and TER of cell monolayers were measured with solution A [140 mM NaCl, 5 mM glucose, 5 mM KCl, 1 mM MgCl_2_, 1 mM CaCl_2_ and 10 mM HEPES-NaOH (pH 7.4)] in the apical side and solution B [70 mM NaCl, 130 mM sucrose, 5 mM glucose, 5 mM KCl, 1 mM MgCl_2_, 1 mM CaCl_2_ and 10 mM HEPES-NaOH (pH 7.4)] in the basal side at 37°C. The *P*_Na_/*P*_Cl_ ratio was calculated using the Goldman–Hodgkin–Katz equation. The values of *P*_Na_ and *P*_Cl_ were then calculated from the TER and *P*_Na_/*P*_Cl_ using the Kimizuka–Koketsu equation [45]. For transcellular transport-inhibitor studies, the epithelia were incubated in solution A in the apical side and solution B in the basal side with 10μM benzamil, 100μM bumetanide and 1mM ouabain in both sides for 30 min at 37°C. *P*_Na_ and *P*_Cl_ were measured before and 30 min after the administration of the inhibitors. N = 3–4 for each experiment.

### Tracer flux

Epithelia grown on filters were incubated in solution A with 0.2 mM fluorescein or FITC-dextran in the basal side for 1 h, and the solutions were collected in the apical side. Fluorescence of the solutions at 518 nm was measured using a fluorescence spectrophotometer (F-4500; Hitachi High-Tech) with an excitation wavelength of 488 nm. The amounts of fluorescein and FITC–dextran were determined by extrapolation from a standard curve of known fluorescein or FITC–dextran concentrations using linear regression. The permeability of fluorescein and FITC-dextran was defined as (dQ/dt)/AC_0_ [36].

### Surface biotinylation assay

We performed a tracer experiment using a primary amine-reactive biotinylation reagent which labels proteins and primary amine-containing macromolecules on a cell surface [23]. Epithelia grown on 12-mm filters were incubated in solution A with 0.01mg/ml EZ-Link Sulfo-NHS-Biotin (Life Technologies) in the basal side for 10 min at 37°C. Then epithelia were fixed with 10% paraformaldehyde for 7 min and pemeabilized with Triton X-100. The bound biotin was detected by alexa fluor 555 streptavidin (S-32355; Life Technologies). The samples were imaged on a Zeiss LSM700 confocal microscope in a similar manner as described in *Immunocytochemistry*.

### Signal inhibitor and activator studies

To examine the effects of signal inhibitors and activators on the doubling time of MDCK I cells, we seeded MDCK I cells at a density of 5.0 × 10^4^ cells/well on a 12-well plate (Falcon, 3.38 cm^2^/well). The cells were grown in the culture medium containing 10μM H89, 10μM genistein, 500nM Go6983, 200μM SQ22536, 10μM forskolin or 500μM IBMX for 48 h. The doubling time was calculated on the assumption that the rate of cell proliferation was constant during the 48 h of the culture. For the measurement of TER and stratification index, epithelia were grown on filters in the culture medium containing the signal inhibitor or activator.

### Statistical analysis

Data are represented as means ± standard error of the mean. Statistical analysis was performed using Student’s *t*-test and Bonferroni correction for multiple comparisons. *P* < 0.05 was considered statistically significant.

## Supporting Information

S1 Fig Quantification of epithelial stratification. To quantitatively evaluate the degree of epithelial stratification, vertical lines were drawn at 20 μm intervals on a vertical section of the epithelial cell sheets, and the cell number on the vertical lines were counted. More than 140 lines were analyzed per sample, and the mean value of the cell number on the lines was defined as stratification index.
(TIF)

S2 Fig Localization of ZO-1 and F-actin under the ‘Apical’ and ‘Basal’ conditions in Caco-2 cells. (A and B) Immunofluorescence microscopy for ZO-1 and F-actin in z-axis plane under the ‘Apical’ (A) and ‘Basal’ (B) conditions in Caco-2 cells. The signals of ZO-1 were observed within the multi-layered Caco-2 cells under the ‘Basal’ condition (*arrows*). *A*, apical side; *B*, basal side. (C and D) Immunofluorescence microscopy for ZO-1 and F-actin in xy plane under the ‘Apical’ (C) and ‘Basal’ (D) conditions in Caco-2 cells. In the middle level of multi-layered Caco-2 cells under the ‘Basal’ condition, the lines of ZO-1 signals were observed with spherical staining of F-actin (*arrows*). (E) Quantification of the signal intensity of ZO-1 within the multilayered epithelia. Scale bars = 10 μm.
(TIF)

S3 Fig Effects of the amount of culture medium on the cell number in MDCK I cells. MDCK I cells were seeded at a density of 2 × 10^5^ cells/cm^2^ on filters, and the cell number was counted with counting chamber after the trypsinization of the cells at each time point. The density of MDCK I cells at four days after the culture under the ‘Increase’ condition was comparable to that under the ‘Apical’ condition.
(TIF)

S4 Fig Debris in the culture supernatant in the microcapillary tubes and quantification of TUNEL signals. (A) The culture supernatant of MDCK I cells under the ‘Apical’ and ‘Basal’ conditions was collected every two days at the exchange of the culture medium, and the supernatant was put into the microcapillary tubes with 0.29 mm inner diameter and centrifuged to pack the debris. Scale bar = 2 mm. (B) Light microscopic images of the packed debris in the culture supernatant. Scale bars = 100 μm for the left panel and 50 μm for the right panel. (C) Quantification of the density of TUNEL signals under the conditions in Fig 9.
(TIF)

S5 Fig Effects of hydrostatic pressure on transcellular transport in MDCK I cells. (A) Effects of the amount of culture medium on TER in MDCK I cells. The TER under the ‘Increase’ condition was comparable to that under the ‘Apical’ condition. (B) Effects of hydrostatic pressure on transepithelial electrical potentials (Vt) in MDCK I cells. Vt was measured at four days after the culture under the ‘Apical’ and ‘Basal’ conditions. A positive Vt represents that an electrical potential in the basal side is higher than that in the apical side. (C) Effects of transcellular transport-inhibitors on *P*_Na_ and *P*_Cl_ under the ‘Apical’ and ‘Basal’ conditions in MDCK I cells. MDCK I cells were cultured under the ‘Apical’ and ‘Basal’ conditions for four days, and the *P*_Na_ and *P*_Cl_ were measured before (−) and 30 min after (+) the administration of 10μM benzamil, 100μM bumetanide and 1mM ouabain in both the apical and basal sides.
(TIF)

S6 Fig Immunofluorescence microscopy for claudin-2 and ZO-1 in MDCK I cells under the ‘Apical’ and ‘Basal’ conditions. Immunofluorescence microscopy for claudin-2 and ZO-1 was performed in MDCK I cells at four days after the culture under the ‘Apical’ and ‘Basal’ conditions. Claudin-2 staining was clearly detected at cell-cell contacts in some regions under the ‘Basal’ condition. Scale bar = 10 μm.
(TIF)

S7 Fig Effects of hydrostatic pressure on TER, *P*_Na_ and *P*_Cl_ in MDCK II cells. (A) Effects of hydrostatic pressure on TER in MDCK II cells. (B) The ratio of *P*_Na_ to *P*_Cl_ (*P*_Na_/*P*_Cl_) under the ‘Apical’ and ‘Basal’ conditions in MDCK II cells. The *P*_Na_/*P*_Cl_ was measured at four days after the culture under the ‘Apical’ and ‘Basal’ conditions. (C) *P*_Na_ and *P*_Cl_ under the ‘Apical’ and ‘Basal’ conditions in MDCK II cells.
(TIF)

S1 Movie Three-dimensional images of ZO-1 signals in the multi-layered MDCK I cells under the ‘Basal’ condition. ZO-1 signals under the ‘Basal’ condition in MDCK I cells were captured by confocal microscopy, and three-dimensional images were constructed from an integration of the confocal scanning images.
(AVI)

S1 Table Volume of the culture medium in the apical and basal sides under the various conditions in this study. Epithelial cells were seeded on 12-mm diameter filters, and the amounts of the culture medium shown in the table were added to the apical and basal sides by a pipette at the exchange of the culture medium.
(TIF)

## References

[1] Balkwill F, Mantovani A (2001) Inflammation and cancer: back to Virchow? Lancet 357: 539–45. 11229684 10.1016/S0140-6736(00)04046-0

[2] Coussens LM, Werb Z (2002) Inflammation and cancer. Nature 420: 860–7. 12490959 10.1038/nature01322 PMC2803035

[3] Mantovani (2010) Molecular pathways linking inflammation and cancer. Curr Mol Med 10: 369–73. 20455855 10.2174/156652410791316968

[4] Vendramini-Costa DB, Carvalho JE (2012) Molecular link mechanisms between inflammation and cancer. Curr Pharm Des 18: 3831–52. 22632748 10.2174/138161212802083707

[5] Heldin CH, Rubin K, Pietras K, Ostman A (2004) High interstitial fluid pressure—an obstacle in cancer therapy. Nat Rev Cancer 4: 806–13. 15510161 10.1038/nrc1456

[6] Diresta GR, Nathan SS, Manoso MW, Casas-Ganem J, Wyatt C, Kubo T, et al (2005) Cell proliferation of cultured human cancer cells are affected by the elevated tumor pressures that exist in vivo. Ann Biomed Eng 33: 1270–80. 16133932 10.1007/s10439-005-5732-9

[7] Lunt SJ, Fyles A, Hill RP, Milosevic M (2008) Interstitial fluid pressure in tumors: therapeutic barrier and biomarker of angiogenesis. Future Oncol 4: 793–802. 10.2217/14796694.4.6.793 19086846

[8] Aung KZ, Pereira BP, Tan PH, Han HC, Nathan SS (2012) Interstitial fluid pressure as an alternate regulator of angiogenesis independent of hypoxia driven HIF-1α in solid tumors. J Orthop Res 30: 2038–45. 10.1002/jor.22154 22622799

[9] Tokuda S, Miyazaki H, Nakajima K, Yamada T, Marunaka Y (2009) Hydrostatic pressure regulates tight junctions, actin cytoskeleton and transcellular ion transport. Biochem Biophys Res Commun 390: 1315–21. 10.1016/j.bbrc.2009.10.144 19879247

[10] Milosevic M, Fyles A, Hedley D, Pintilie M, Levin W, Manchul L, et al (2001) Interstitial fluid pressure predicts survival in patients with cervix cancer independent of clinical prognostic factors and tumor oxygen measurements. Cancer Res 61: 6400–5. 11522633

[11] Barker G, Simmons NL (1981) Identification of two strains of cultured canine renal epithelial cells (MDCK cells) which display entirely different physiological properties. Q J Exp Physiol 66: 61–72. 6911762 10.1113/expphysiol.1981.sp002529

[12] Farquhar MG, Palade GE (1963) Junctional complexes in various epithelia. J Cell Biol 17: 375–412. 13944428 10.1083/jcb.17.2.375 PMC2106201

[13] Dragsten PR, Blumenthal R, Handler JS (1981) Membrane asymmetry in epithelia: is the tight junction a barrier to diffusion in the plasma membrane? Nature 294: 718–22. 7322203 10.1038/294718a0

[14] Staehelin LA (1973) Further observations on the fine structure of freeze-cleaved tight junctions. J Cell Sci 13: 763–86. 4203962 10.1242/jcs.13.3.763

[15] Stevenson BR, Siliciano JD, Mooseker MS, Goodenough DA (1986) Identification of ZO-1: a high molecular weight polypeptide associated with the tight junction (zonula occludens) in a variety of epithelia. J Cell Biol 103: 755–66. 3528172 10.1083/jcb.103.3.755 PMC2114282

[16] Furuse M, Hirase T, Itoh M, Nagafuchi A, Yonemura S, Tsukita S, et al (1993) Occludin: a novel integral membrane protein localizing at tight junctions. J Cell Biol 123: 1777–88. 8276896 10.1083/jcb.123.6.1777 PMC2290891

[17] Furuse M, Fujita K, Hiiragi T, Fujimoto K, Tsukita S (1998) Claudin-1 and -2: novel integral membrane proteins localizing at tight junctions with no sequence similarity to occludin. J Cell Biol 141: 1539–50. 9647647 10.1083/jcb.141.7.1539 PMC2132999

[18] Stevenson BR, Anderson JM, Goodenough DA, Mooseker MS (1988) Tight junction structure and ZO-1 content are identical in two strains of Madin-Darby canine kidney cells which differ in transepithelial resistance. J Cell Biol 107: 2401–8. 3058723 10.1083/jcb.107.6.2401 PMC2115690

[19] Furuse M, Furuse K, Sasaki H, Tsukita S (2001) Conversion of zonulae occludentes from tight to leaky strand type by introducing claudin-2 into Madin-Darby canine kidney I cells. J Cell Biol 153: 263–72. 11309408 10.1083/jcb.153.2.263 PMC2169456

[20] Wu CJ, Mannan P, Lu M, Udey MC (2013) Epithelial cell adhesion molecule (EpCAM) regulates claudin dynamics and tight junctions. J Biol Chem 288: 12253–68. 10.1074/jbc.M113.457499 23486470 PMC3636909

[21] Claude P, Goodenough DA (1973) Fracture faces of zonulae occludentes from "tight" and "leaky" epithelia. J Cell Biol 58: 390–400. 4199658 10.1083/jcb.58.2.390 PMC2109050

[22] Powell DW (1981) Barrier function of epithelia. Am J Physiol 241: G275–88. 7032321 10.1152/ajpgi.1981.241.4.G275

[23] Chen Y, Merzdorf C, Paul DL, Goodenough DA (1997) COOH terminus of occludin is required for tight junction barrier function in early Xenopus embryos. J Cell Biol 138: 891–9. 9265654 10.1083/jcb.138.4.891 PMC2138038

[24] Gratzner HG (1982) Monoclonal antibody to 5-bromo- and 5-iododeoxyuridine: A new reagent for detection of DNA replication. Science 218:474–5. 7123245 10.1126/science.7123245

[25] Eagle H, Levine EM (1967) Growth regulatory effects of cellular interaction. Nature 213: 1102–6. 6029791 10.1038/2131102a0

[26] Puliafito A, Hufnagel L, Neveu P, Streichan S, Sigal A, Fygenson DK, et al (2012) Collective and single cell behavior in epithelial contact inhibition. Proc Natl Acad Sci U S A 109: 739–44. 10.1073/pnas.1007809109 22228306 PMC3271933

[27] Kerr JF, Wyllie AH, Currie AR (1972) Apoptosis: a basic biological phenomenon with wide-ranging implications in tissue kinetics. Br J Cancer. 26: 239–57. 4561027 10.1038/bjc.1972.33 PMC2008650

[28] Gavrieli Y, Sherman Y, Ben-Sasson SA (1992) Identification of programmed cell death in situ via specific labeling of nuclear DNA fragmentation. J Cell Biol 119: 493–501. 1400587 10.1083/jcb.119.3.493 PMC2289665

[29] Yu AS, Cheng MH, Angelow S, Günzel D, Kanzawa SA, Schneeberger EE, et al (2009) Molecular basis for cation selectivity in claudin-2-based paracellular pores: identification of an electrostatic interaction site. J Gen Physiol 133: 111–27. 10.1085/jgp.200810154 19114638 PMC2606938

[30] Reuss L (2001) Tight junction permeability to ions and water In: Anderson JM, Cereijido M, editors. Tight junctions, Second Edition CRC Press Boca Raton pp. 61–88.

[31] Anderson JM, Van Itallie CM (2009) Physiology and function of the tight junction. Cold Spring Harb Perspect Biol 1: a002584 10.1101/cshperspect.a002584 20066090 PMC2742087

[32] Van Itallie CM, Anderson JM (2006) Claudins and epithelial paracellular transport. Annu Rev Physiol 68: 403–29. 16460278 10.1146/annurev.physiol.68.040104.131404

[33] Günzel D, Yu AS (2013) Claudins and the modulation of tight junction permeability. Physiol Rev 93: 525–69. 10.1152/physrev.00019.2012 23589827 PMC3768107

[34] Amasheh S, Meiri N, Gitter AH, Schöneberg T, Mankertz J, Schulzke JD, et al (2002) Claudin-2 expression induces cation-selective channels in tight junctions of epithelial cells. J Cell Sci 115: 4969–76. 12432083 10.1242/jcs.00165

[35] Van Itallie CM, Fanning AS, Anderson JM (2003) Reversal of charge selectivity in cation or anion-selective epithelial lines by expression of different claudins. Am J Physiol Renal Physiol 285: F1078–84 13129853 10.1152/ajprenal.00116.2003

[36] Tokuda S, Furuse M (2015) Claudin-2 knockout by TALEN-mediated gene targeting in MDCK cells: claudin-2 independently determines the leaky property of tight junctions in MDCK cells. PLoS One 10: e0119869 10.1371/journal.pone.0119869 25781928 PMC4363821

[37] Van Itallie CM, Holmes J, Bridges A, Gookin JL, Coccaro MR, Proctor W, et al (2008) The density of small tight junction pores varies among cell types and is increased by expression ofclaudin-2. J Cell Sci 121: 298–305. 10.1242/jcs.021485 18198187

[38] Michel CC, Curry FE (1999) Microvascular permeability. Physiol Rev 79: 703–61. 10390517 10.1152/physrev.1999.79.3.703

[39] Turksen K, Troy TC (2011) Junctions gone bad: claudins and loss of the barrier in cancer. Biochim Biophys Acta 1816: 73–9. 10.1016/j.bbcan.2011.04.001 21515339

[40] Weinberg RA (2013) The biology of cancer, Second Edition Garland Science.

[41] Itoh M, Yonemura S, Nagafuchi A, Tsukita S, Tsukita S (1991) A 220-kD undercoat-constitutive protein: its specific localization at cadherin-based cell-cell adhesion sites. J Cell Biol 115: 1449–62. 1955485 10.1083/jcb.115.5.1449 PMC2289222

[42] Saitou M, Ando-Akatsuka Y, Itoh M, Furuse M, Inazawa J, Fujimoto K, et al (1997) Mammalian occludin in epithelial cells: its expression and subcellular distribution. Eur J Cell Biol 73: 222–31. 9243183

[43] Furuse M, Sasaki H, Tsukita S (1999) Manner of interaction of heterogeneous claudin species within and between tight junction strands. J Cell Biol 147: 891–903. 10562289 10.1083/jcb.147.4.891 PMC2156154

[44] Tokuda S, Higashi T, Furuse M (2014) ZO-1 knockout by TALEN-mediated gene targeting in MDCK cells: involvement of ZO-1 in the regulation of cytoskeleton and cell shape. PLoS One 9: e104994 10.1371/journal.pone.0104994 25157572 PMC4144852

[45] Kimizuka H, Koketsu K (1964) Ion transport through cell membrane. J Theor Biol 6: 290–305. 5875308 10.1016/0022-5193(64)90035-9

