# Supplementary figures and images for: Effects of Hydrostatic Pressure on Carcinogenic Properties of Epithelia
Source: PLoS One. 2015 Dec 30;10(12):e0145522. doi: 10.1371/journal.pone.0145522 (PMC4696811; doi:10.1371/journal.pone.0145522)

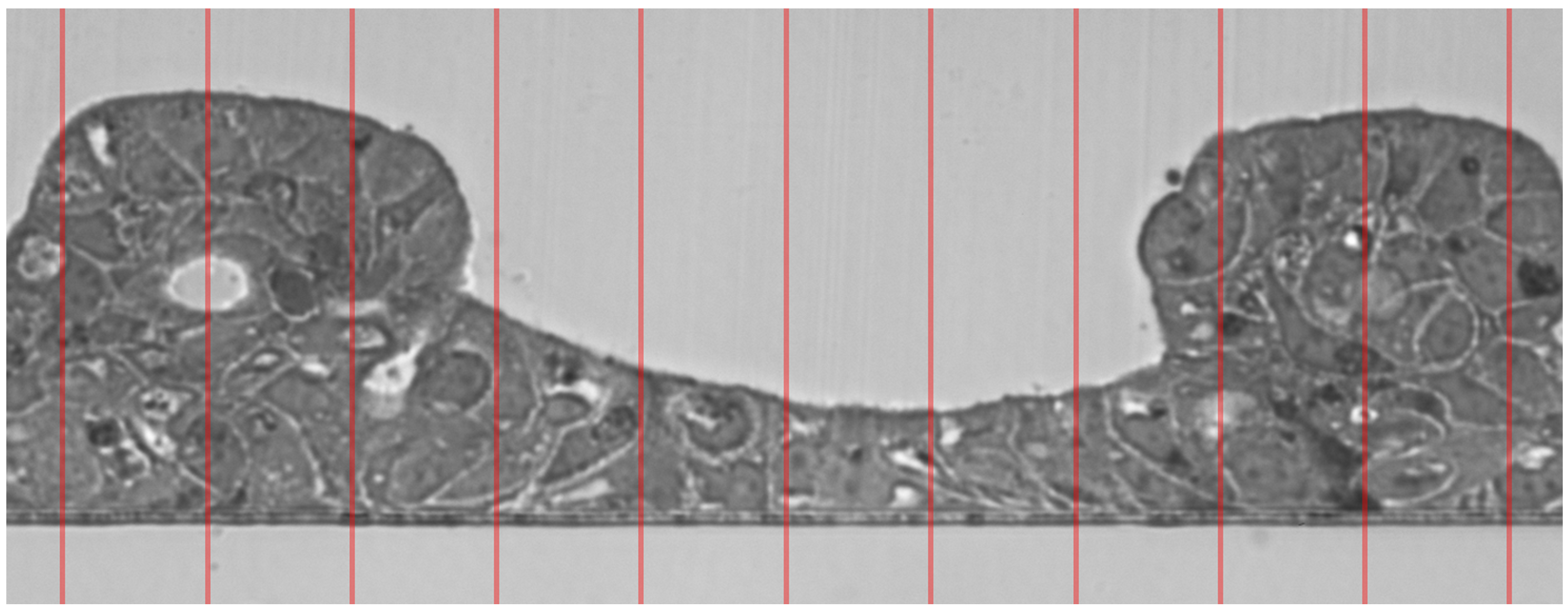

Supplement: S1 Fig — To quantitatively evaluate the degree of epithelial stratification, vertical lines were drawn at 20 μm intervals on a vertical section of the epithelial cell sheets, and the cell number on the vertical lines were counted. More than 140 lines were analyzed per sample, and the mean value of the cell number on the lines was defined as stratification index. (TIF) [file pone.0145522.s001.tif]

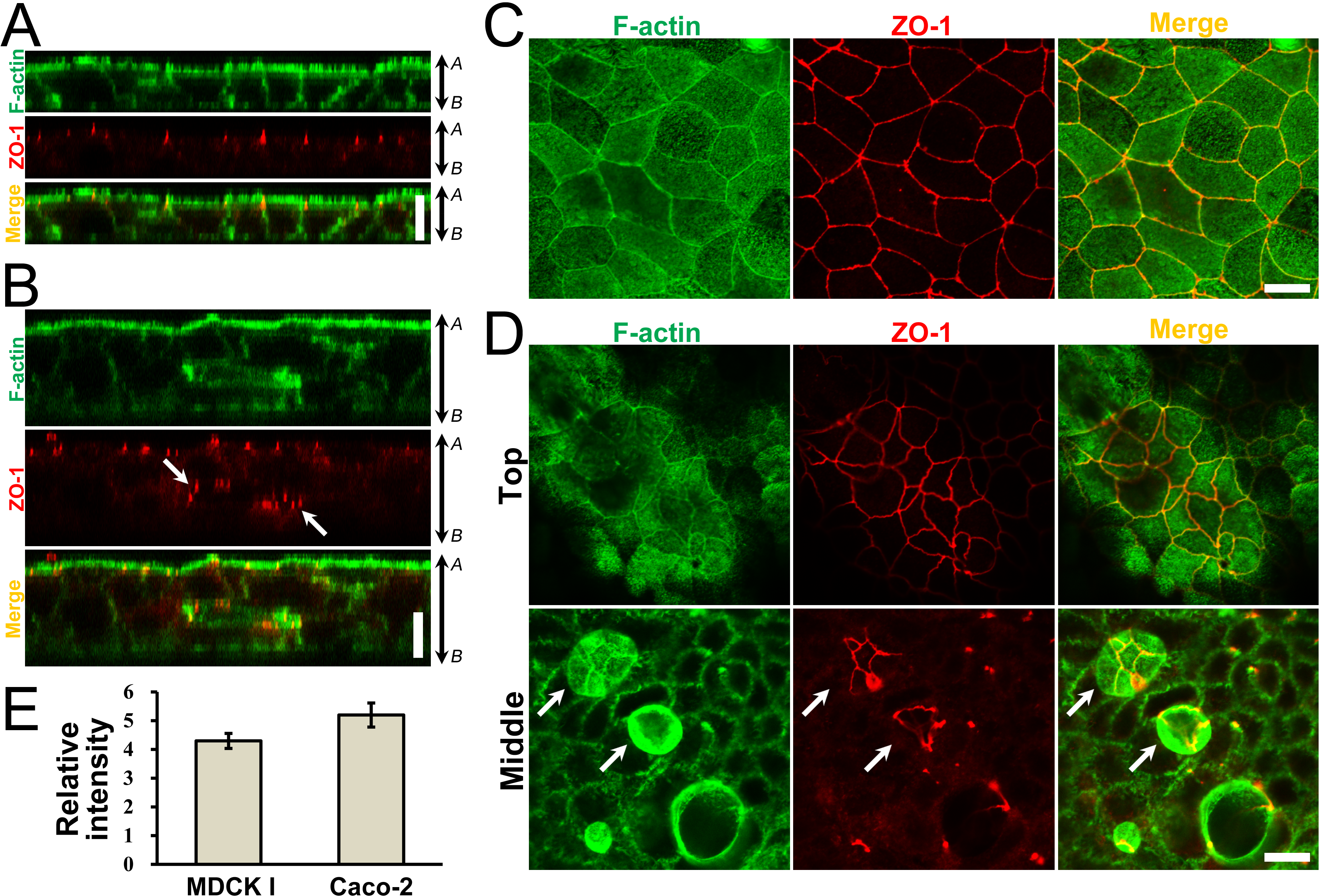

Supplement: S2 Fig — (A and B) Immunofluorescence microscopy for ZO-1 and F-actin in z-axis plane under the ‘Apical’ (A) and ‘Basal’ (B) conditions in Caco-2 cells. The signals of ZO-1 were observed within the multi-layered Caco-2 cells under the ‘Basal’ condition (arrows). A, apical side; B, basal side. (C and D) Immunofluorescence microscopy for ZO-1 and F-actin in xy plane under the ‘Apical’ (C) and ‘Basal’ (D) conditions in Caco-2 cells. In the middle level of multi-layered Caco-2 cells under the ‘Basal’ condition, the lines of ZO-1 signals were observed with spherical staining of F-actin (arrows). (E) Quantification of the signal intensity of ZO-1 within the multilayered epithelia. Scale bars = 10 μm. (TIF) [file pone.0145522.s002.TIF]

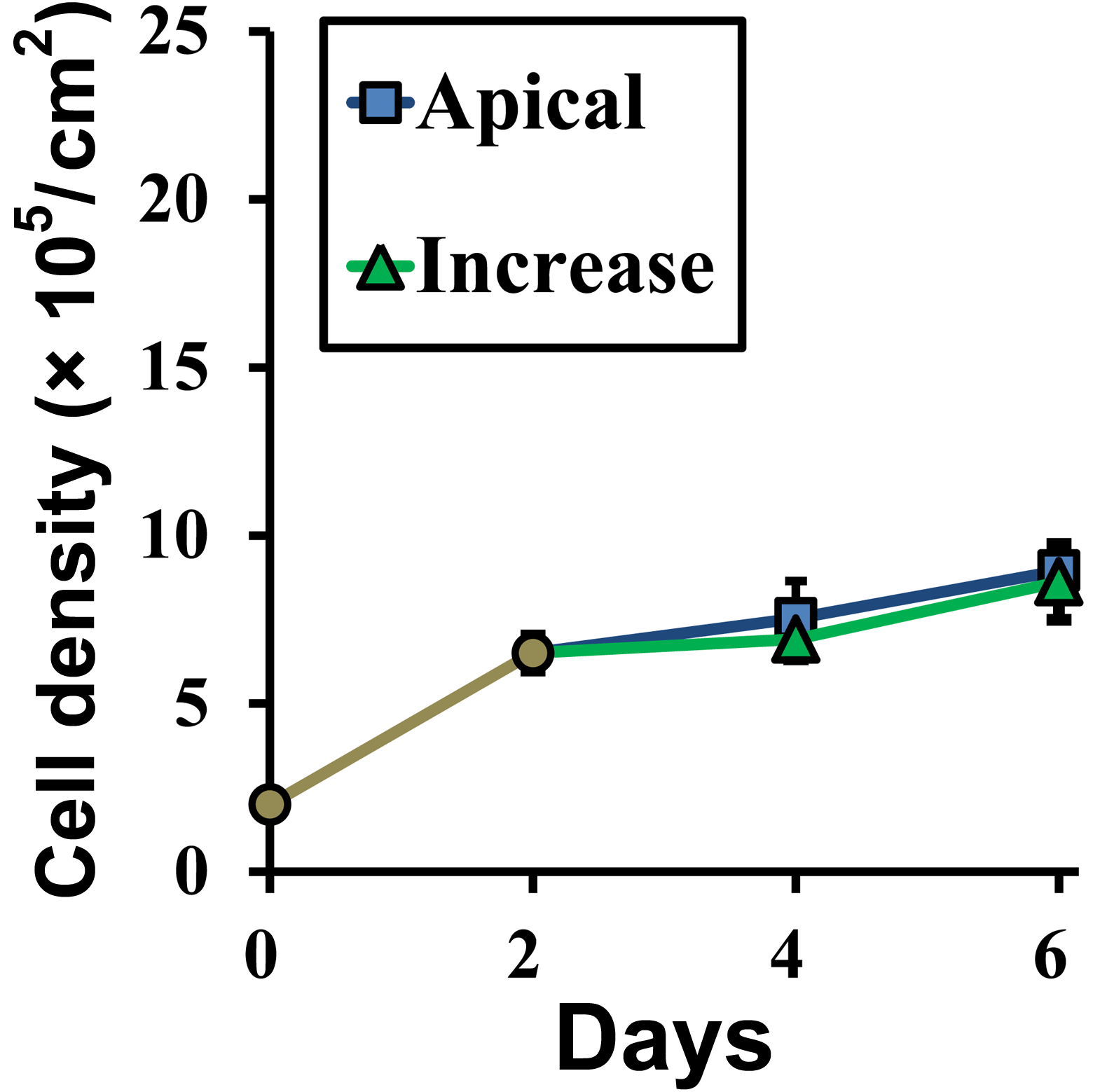

Supplement: S3 Fig — MDCK I cells were seeded at a density of 2 × 10^5^ cells/cm2 on filters, and the cell number was counted with counting chamber after the trypsinization of the cells at each time point. The density of MDCK I cells at four days after the culture under the ‘Increase’ condition was comparable to that under the ‘Apical’ condition. (TIF) [file pone.0145522.s003.TIF]

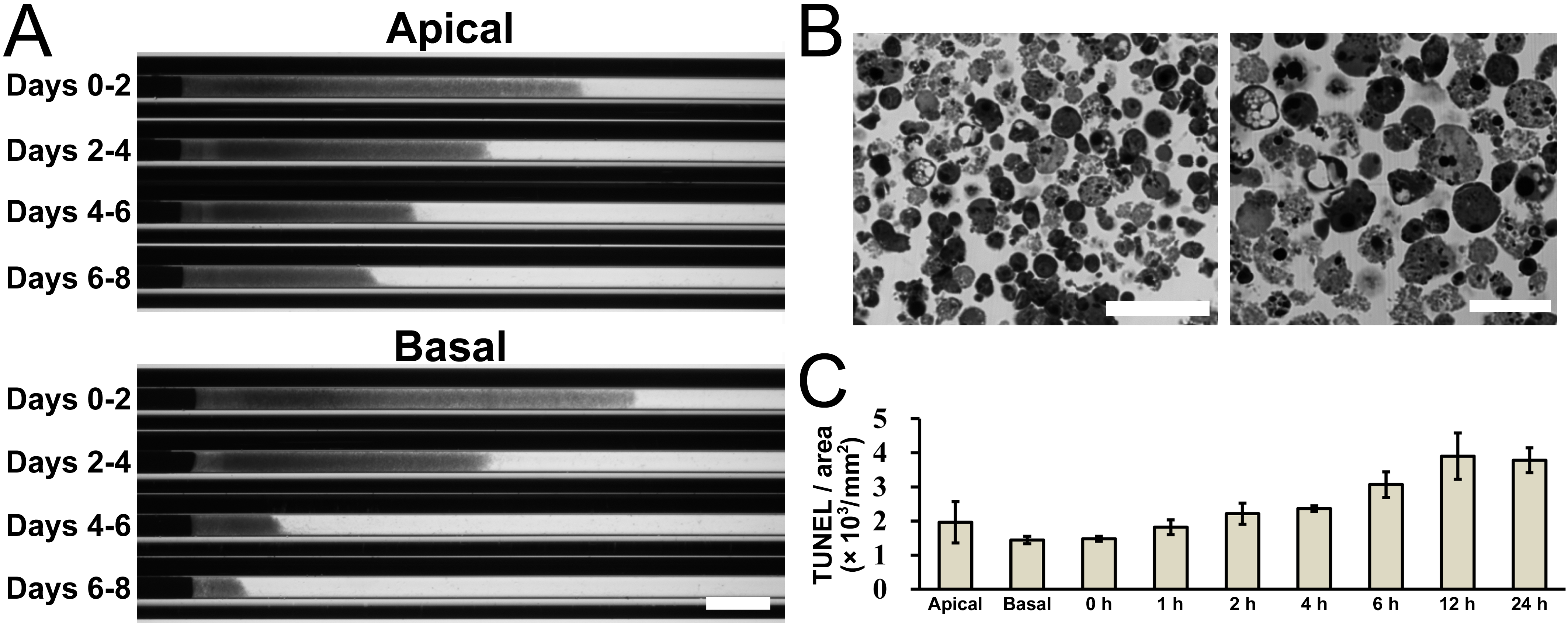

Supplement: S4 Fig — (A) The culture supernatant of MDCK I cells under the ‘Apical’ and ‘Basal’ conditions was collected every two days at the exchange of the culture medium, and the supernatant was put into the microcapillary tubes with 0.29 mm inner diameter and centrifuged to pack the debris. Scale bar = 2 mm. (B) Light microscopic images of the packed debris in the culture supernatant. Scale bars = 100 μm for the left panel and 50 μm for the right panel. (C) Quantification of the density of TUNEL signals under the conditions in Fig 9. (TIF) [file pone.0145522.s004.tif]

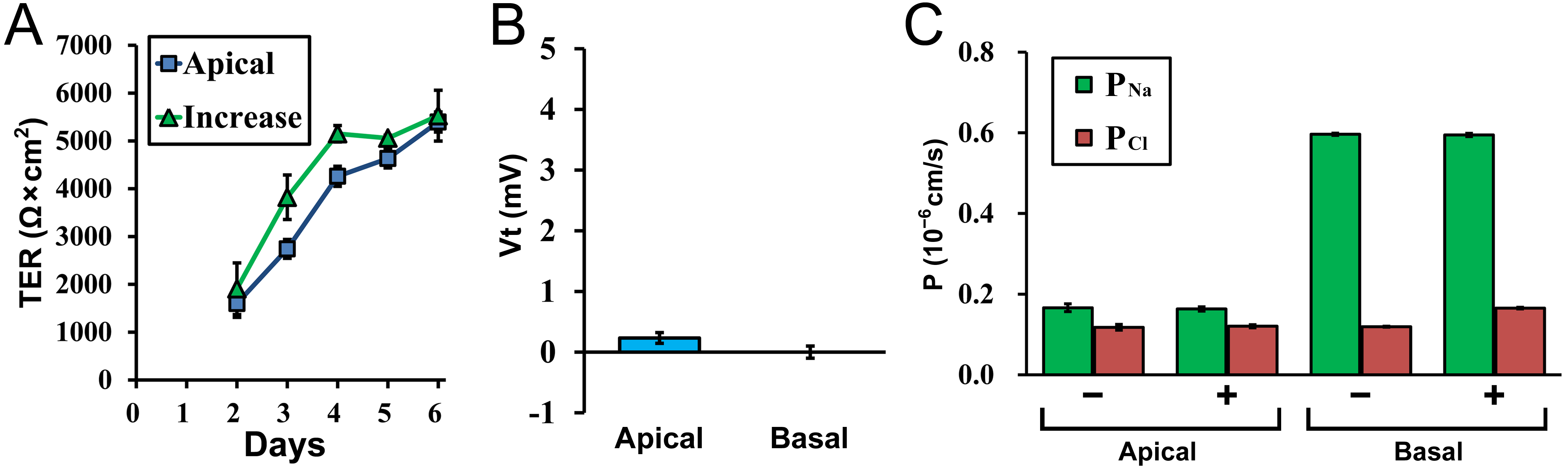

Supplement: S5 Fig — (A) Effects of the amount of culture medium on TER in MDCK I cells. The TER under the ‘Increase’ condition was comparable to that under the ‘Apical’ condition. (B) Effects of hydrostatic pressure on transepithelial electrical potentials (Vt) in MDCK I cells. Vt was measured at four days after the culture under the ‘Apical’ and ‘Basal’ conditions. A positive Vt represents that an electrical potential in the basal side is higher than that in the apical side. (C) Effects of transcellular transport-inhibitors on P Na and P Cl under the ‘Apical’ and ‘Basal’ conditions in MDCK I cells. MDCK I cells were cultured under the ‘Apical’ and ‘Basal’ conditions for four days, and the P Na and P Cl were measured before (−) and 30 min after (+) the administration of 10μM benzamil, 100μM bumetanide and 1mM ouabain in both the apical and basal sides. (TIF) [file pone.0145522.s005.tif]

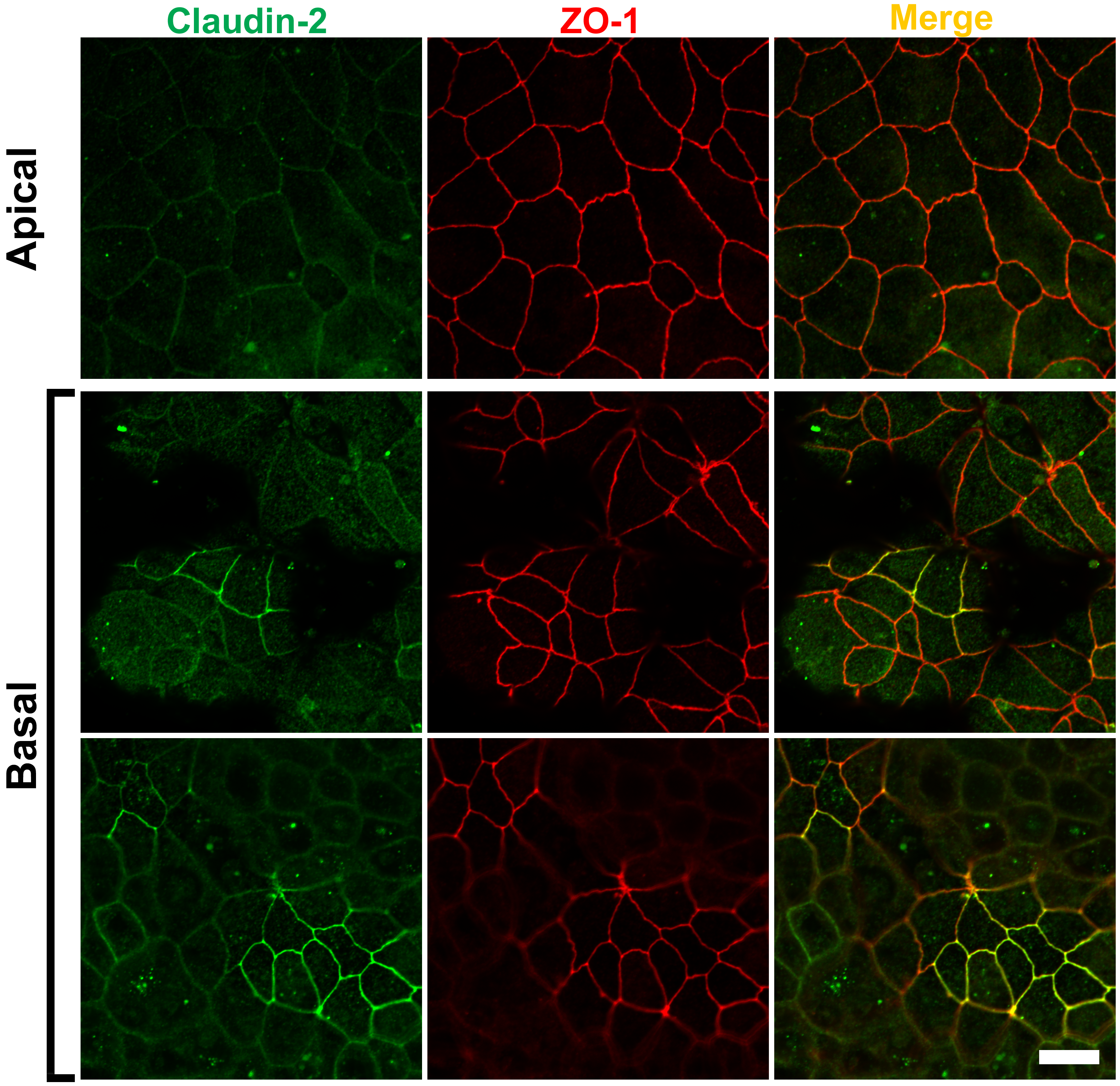

Supplement: S6 Fig — Immunofluorescence microscopy for claudin-2 and ZO-1 was performed in MDCK I cells at four days after the culture under the ‘Apical’ and ‘Basal’ conditions. Claudin-2 staining was clearly detected at cell-cell contacts in some regions under the ‘Basal’ condition. Scale bar = 10 μm. (TIF) [file pone.0145522.s006.tif]

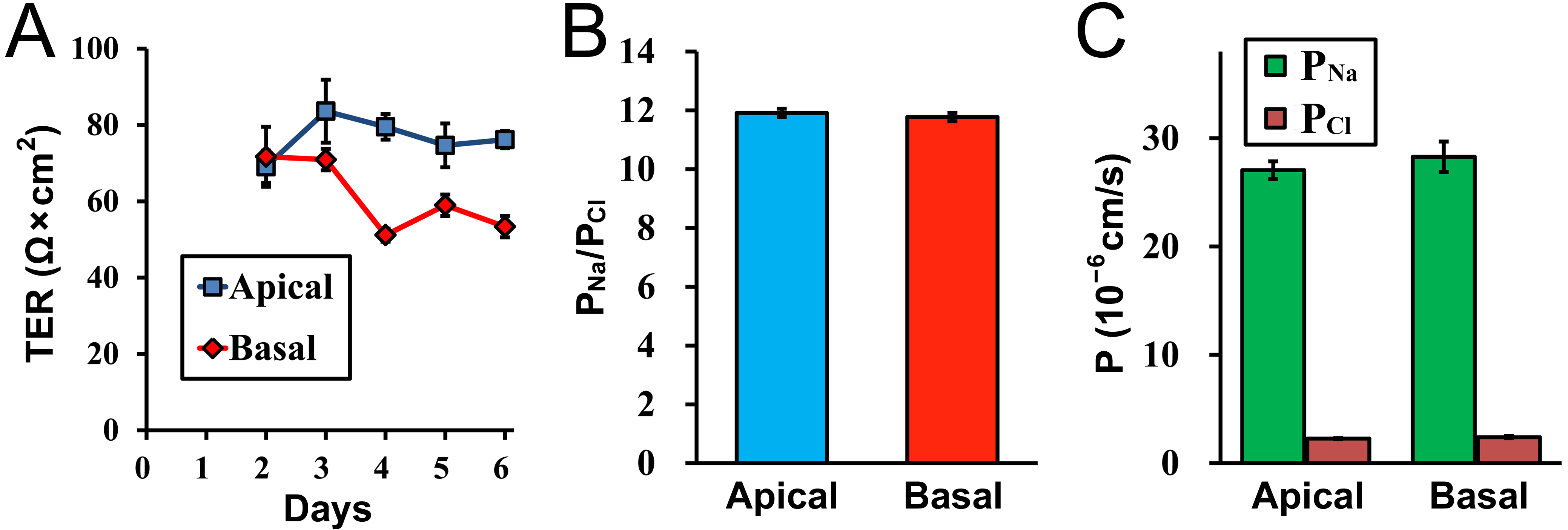

Supplement: S7 Fig — (A) Effects of hydrostatic pressure on TER in MDCK II cells. (B) The ratio of P Na to P Cl (P Na/P Cl) under the ‘Apical’ and ‘Basal’ conditions in MDCK II cells. The P Na/P Cl was measured at four days after the culture under the ‘Apical’ and ‘Basal’ conditions. (C) P Na and P Cl under the ‘Apical’ and ‘Basal’ conditions in MDCK II cells. (TIF) [file pone.0145522.s007.TIF]

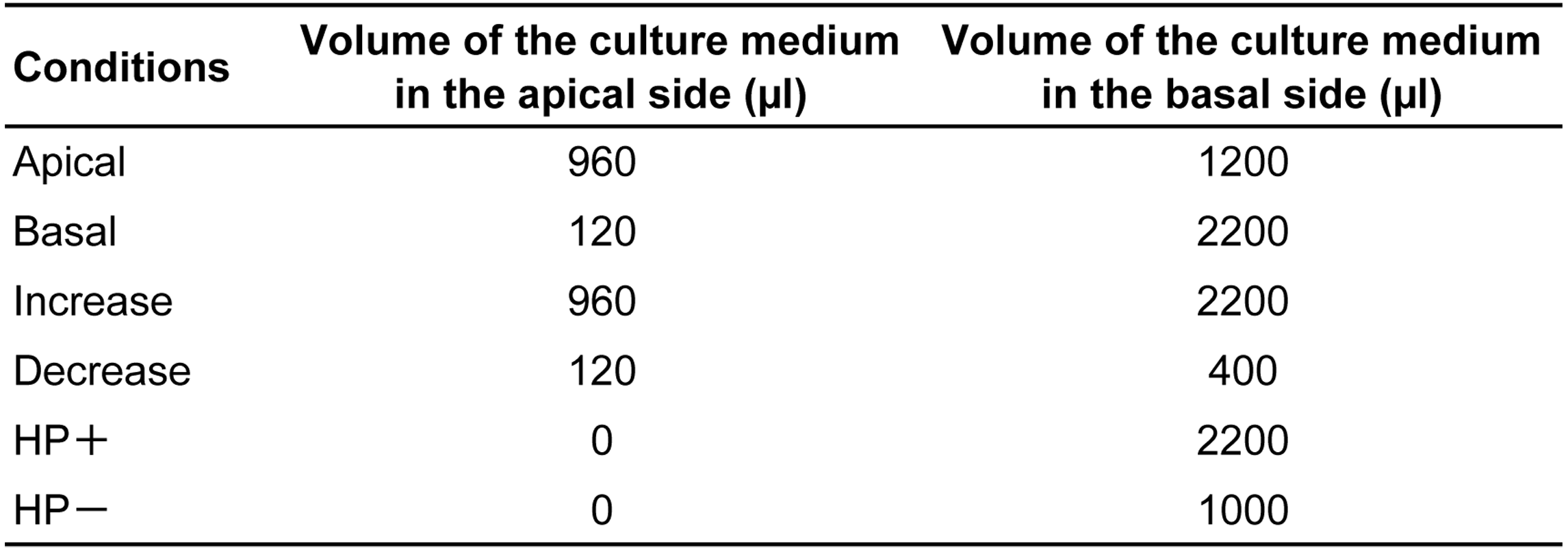

Supplement: S1 Table — Epithelial cells were seeded on 12-mm diameter filters, and the amounts of the culture medium shown in the table were added to the apical and basal sides by a pipette at the exchange of the culture medium. (TIF) [file pone.0145522.s009.TIF]
